# Hexamethylbenzene elimination enables the generation of transient, sterically unhindered multiply bonded boron species†

**DOI:** 10.1039/d5sc02645h

**Published:** 2025-05-19

**Authors:** Chonghe Zhang, Philipp Dabringhaus, Bi Youan E. Tra, Robert J. Gilliard, Christopher C. Cummins

**Affiliations:** a Department of Chemistry, Massachusetts Institute of Technology Cambridge Massachusetts 02139 USA ccummins@mit.edu gilliard@mit.edu

## Abstract

We present a method for the generation of boron-containing unsaturated small molecules *via* hexamethylbenzene elimination. The fragmentation precursors are obtained through bond insertion into phenyl boranorbornadiene (PhB(C_6_Me_6_), 1). Compound 1 undergoes 1,1-insertion with 2,6-xylyl isocyanide, affording a boron-doped bicyclo[2.2.2]octa-2,5-diene 2. Heating 2 in toluene results in the formation of a base-stabilized boraketenimine PhB(CNxyl)_2_ (*i.e.*, borylene diisocyanide) as an intermediate *via* retro-Diels–Alder reaction. Surprisingly, PhB(CNxyl)_2_ dimerizes to give a boron-doped 6-membered ring (PhB)_2_C_4_(CNxyl)_6_4. The reaction of 1 with trimethylamine *N*-oxide and phenyl azide yields triphenyl boroxine and a BN_4_ ring, respectively, implying the involvement of transient oxoborane (PhB≡O) and iminoborane intermediates (PhB≡NPh), respectively. Furthermore, boranorbornadiene also undergoes 2,3-insertion with mesityl isocyanate (MesNCO), affording a fused 6/5-membered heterocycle 11. This insertion profile is analogous to the insertion of phenyl azide into 1.

## Abstract 2

We present a method for the generation of boron-containing unsaturated small molecules *via* hexamethylbenzene elimination.

## Graphical abstract

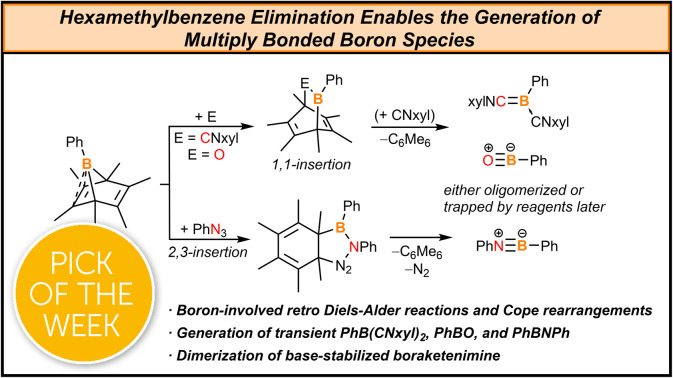

## Introduction

**Fig. 1:**
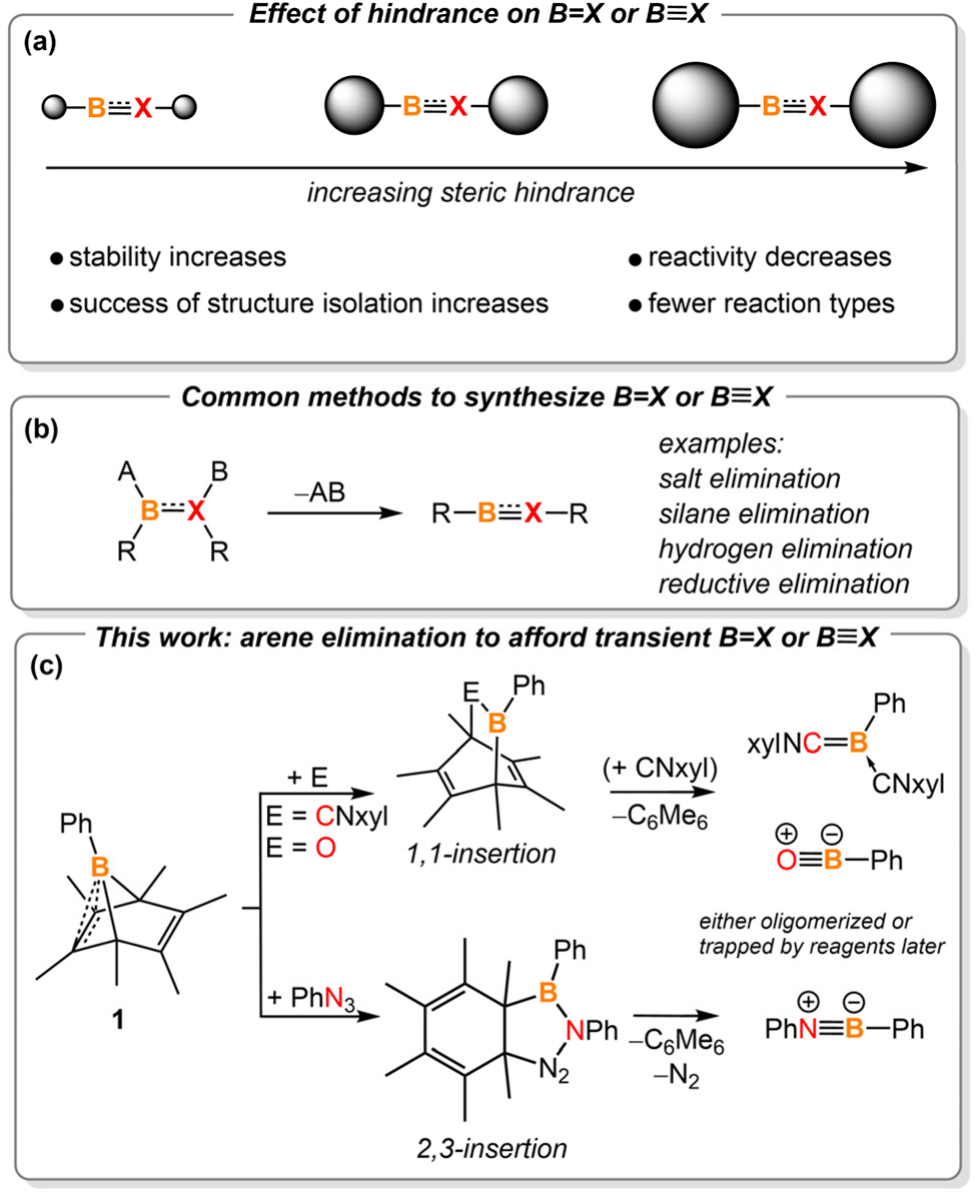
(a) Effects of steric hindrance on multiply bonded boron species; (b) common methods to synthesize boron-containing double bonds or triple bonds; (c) hexamethylbenzene elimination enables the generation of boron unsaturated bonds (this work).

Molecules with boron-containing unsaturated bonds are known as key synthetic intermediates for accessing boron-doped heterocycles, which are important in the fields of catalysis,^1^ medicinal chemistry,^2^ luminescent materials,^3^ and chemical sensors.^4^ In addition, they are fascinating mediators for small molecule activation due to the intrinsic instability of the bonds compared to their carbon-based counterparts.^5^ The synthesis and reactivity profiles of compounds containing boron–second-row element unsaturated bonds are well established. They are commonly isolated by attaching kinetically stabilizing bulky substituents to the boron moiety (Fig. 1a).^5*i*,6^ The enhanced steric hindrance prevents the target species from oligomerizing or reacting with reagents used in the synthesis.5f,g However, the steric bulk also decreases the reactivity of these molecules, limiting the discovery of new reaction types.^7^ Thus, developing methods to cleanly generate transient, non-sterically hindered small molecules with unsaturated boron multiple bonds is the key to unlocking reactivity patterns that are currently hidden.

Removing two substituents from boron and its adjacent atom is the most common method to construct boron unsaturated bonds (*i.e.*, elimination, Fig. 1b).5i,6b,8 While the eliminated substituent on boron is often a halide or hydride, the adjacent atom's substituent varies depending on its electronegativity. For example, salt or silane elimination often occurs in the construction of compounds containing BN and BO unsaturated bonds.^6*b*,8*b*,9^ Recently, we used arenes, specifically benzene and anthracene, as the leaving group to synthesize diazoborane^10^ and transient borylene intermediates.^11^ Such arene extrusion reactions represent a mild approach to elimination processes, where aromatization would provide the extra driving force necessary for boron-centered elimination chemistry. We postulated that such arene extrusion reactions could also be applied to synthesizing transient, sterically unhindered multiply bonded boron species.

Boranorbornadienes are boron analogues of norbornadienes.^12^ In 1988, Fagan reported the synthesis of phenyl-substituted hexamethyl-boranorbornadiene PhB(C_6_Me_6_). Recently, we reported the synthesis of the halo-substituted versions (RB(C_6_Me_6_), R = Cl, Br), which were used to generate carbene-ligated haloborylenes as reactive intermediates.^11^ Herein, we report the generation of transient base-stabilized boraketenimine (PhB(CNxyl)_2_), oxoborane (PhB≡O), and iminoborane (PhB≡NPh) *via* hexamethylbenzene elimination (Fig. 1c). Dimerization of a boraketenimine is reported here for the first time, illustrating the privilege of non-sterically hindered boron unsaturated species. In addition, boron-involved retro-Diels–Alder reactions and Cope rearrangements are also disclosed in this work.

## Results and discussion

### Generating a transient boraketenimine

**Scheme 1:**
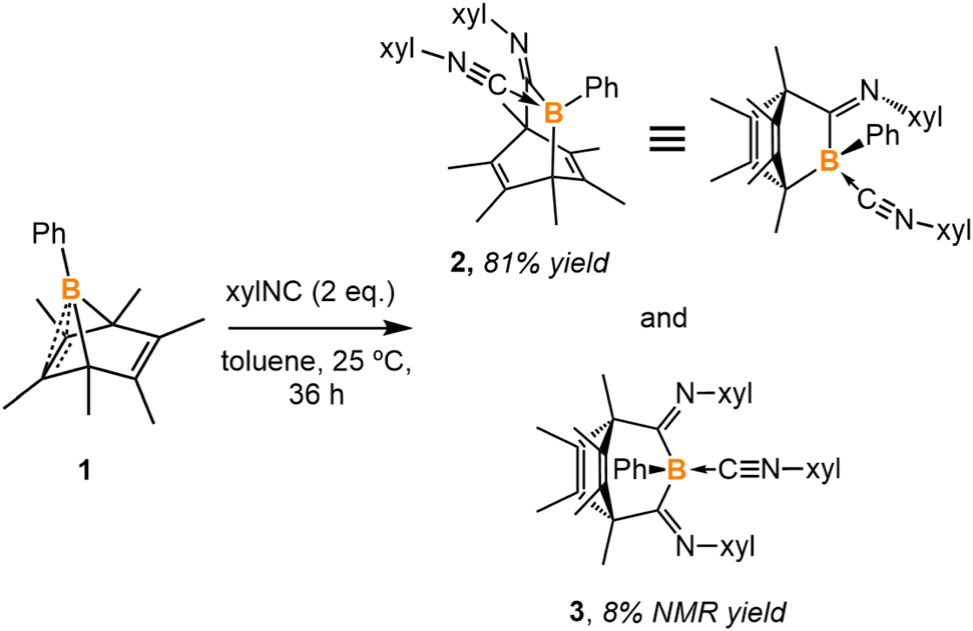
Reaction of PhB(C_6_Me_6_) (1) with 2 equivalents of 2,6-xylyl isocyanide (xylNC) affords 2 in 81% yield and 3 as a side product.

**Fig. 2:**
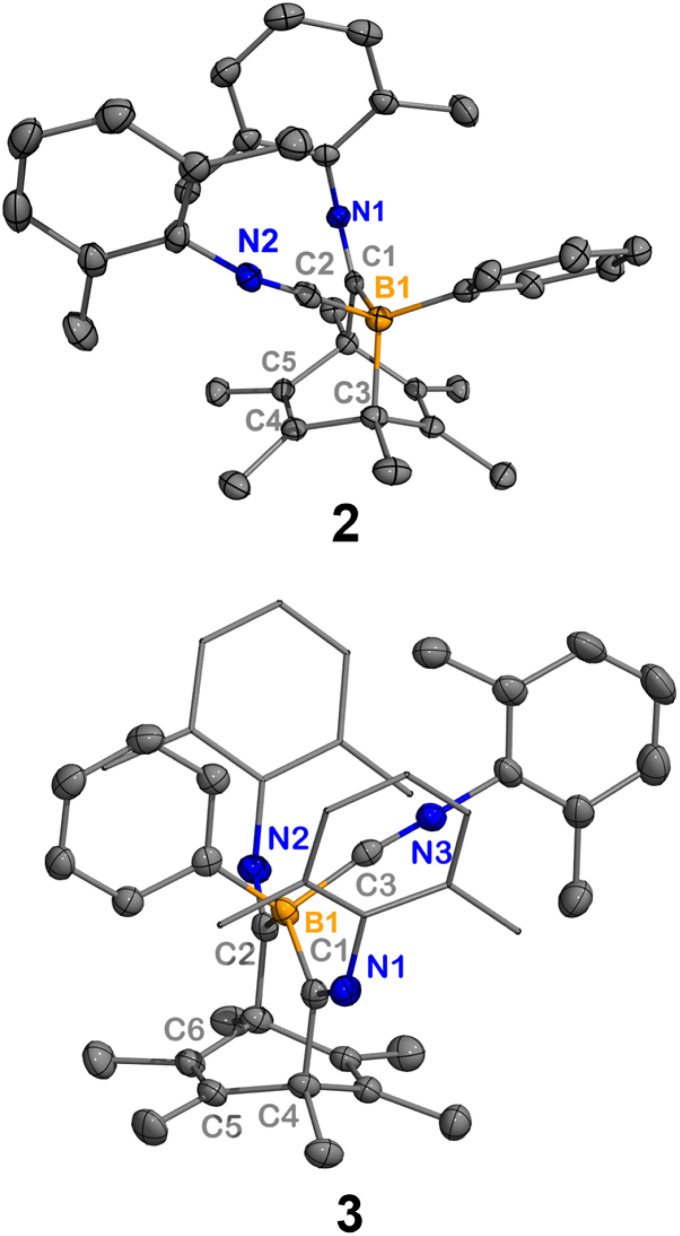
Molecular structures of 2 and 3. Hydrogen atoms have been omitted for clarity. Thermal ellipsoids are drawn at the 50% probability level. Selected bond lengths [Å] and angles [°]: 2: B1–C1 1.638(2), B1–C2 1.588(2), C1–N1 1.281(2), C2–N2 1.159(2), B1–C3 1.679(2), C3–C4 1.528(2), C4–C5 1.334(2), B1–C1–N1 131.1(1), B1–C2–N2 177.3(1); 3: B1–C1 1.673(3), B1–C2 1.671(3), B1–C3 1.593(3), C1–N1 1.290(3), C2–N2 1.280(2), C3–N3 1.154 (3), C4–C5 1.529(3), C5–C6 1.329(3), B1–C1–N1 127.1(2), B1–C2–N2 126.7(2), B1–C3–N3 172.8(2).

In the first experiment, we targeted the preparation of a precursor for a boraketenimine containing a B=C double bond.^13^ It should be mentioned that the stabilization of boraketenimines typically demands bulky aryl substituents.^14^ The reaction of PhB(C_6_Me_6_) (1) with 2,6-xylyl isocyanide (xylNC) (2 equiv.) in toluene afforded two new boron species according to the ^11^B NMR spectrum, one with a singlet at −17.8 ppm and one with a singlet at −13.5 ppm (Scheme 1). The reaction was monitored by NMR spectroscopy, and it required three days to go to completion. Storing the concentrated reaction mixture at −35 °C yielded block-shaped crystals and a few plate-shaped crystals. Single-crystal X-ray diffraction (SC-XRD) analysis of the block-shaped crystal revealed a boron-doped bicyclo[2.2.2]octa-2,5-diene 2 (Fig. 2). It is rationalized that compound 2 is generated from 1 through the insertion of an isocyanide followed by the coordination of another isocyanide to the boron. In the solid-structure of 2, the B1–C2 bond (1.588(2) Å) is remarkably shorter than the B1–C1 and B1–C3 single bonds (1.638(2) Å, 1.679(2) Å, respectively). The C1–N1 and C2–N2 distances are 1.281(2) Å and 1.159(2) Å, which fall within the range of C=N double bonds and C≡N triple bonds, respectively.^15^ SC-XRD analysis of the plate-shaped crystal revealed a boron-doped bicyclo[3.2.2]nona-6,8-diene 3, which represents the product of a double isocyanide insertion into 1. The B1–C1 and B1–C2 distances are 1.673(3) Å and 1.671(3) Å, respectively, comparable to the B1–C1 distance in 2.

Despite various attempts, the formation of the side-product 3 could not be prevented, nor could 3 be generated quantitatively. Increasing the equivalents of xylNC (*e.g.*, 4 eq.) led to a notable enhancement in the yield of 3 (15% NMR yield). However, extension of the reaction time did not result in the conversion of 2 to 3. The treatment of 1 with equimolar xylNC at room temperature resulted in the complete consumption of xylNC and half consumption of boranorbornadiene 1, suggesting that the isocyanide insertion step is the rate-determining step, and the coordinated isocyanide becomes unreactive.

**Fig. 3:**
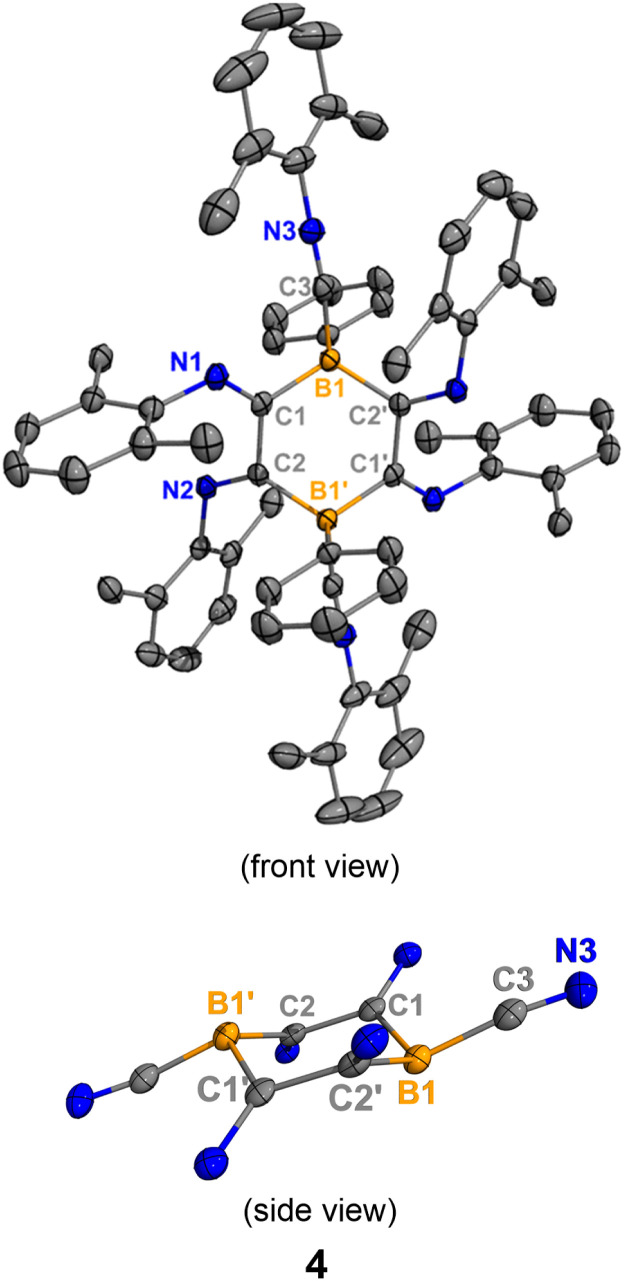
Molecular structure of 4. Hydrogen atoms and all dimethylphenyl groups in the bottom representation have been omitted for clarity to show the central diboracyclohexane unit. Thermal ellipsoids are drawn at the 50% probability level. Selected bond lengths [Å] and angles [°]: B1–C1 1.642(2), C1–C2 1.520(2), C1–N1 1.282(2), B1–C3 1.599(2), C3–N3 1.151(2), B1–C1–C2 115.5(1), B1–C3–N3 168.5(2), N1–C1–C2 126.0(1), B1–C1–C2–B1′−53.6(2).

Heating a benzene solution of 2 at 80 °C overnight led to a color change from yellow to dark red and the precipitation of yellow crystals. The ^1^H NMR spectrum of the crude reaction mixture indicated the formation of free hexamethylbenzene (2.13 ppm in C_6_D_6_). SC-XRD analysis of the yellow crystals yielded the structure of a tetraiminyl-1,4-diboracyclohexane 4. In the molecular structure of 4, the B1–C1 and B1′–C2 distances are measured to be 1.603(2) Å and 1.640(2) Å, respectively, which fall within the range of B–C single bonds (Fig. 3). The C1–C2 distance of 1.502(2) Å lies within the range of C–C single bonds.^15,16^ The geometry of the B1–C1–C2–B1′–C1′–C2′ 6-membered ring is comparable to the chair conformation of cyclohexane. Compound 4 is insoluble in common solvents such as benzene, toluene, dichloromethane, and THF, preventing NMR spectroscopic analysis at room temperature. However, heating 4 in toluene-D_8_ at 105 °C allowed for the observation of a singlet at −10.9 ppm in the ^11^B NMR spectrum.

**Scheme 2:**
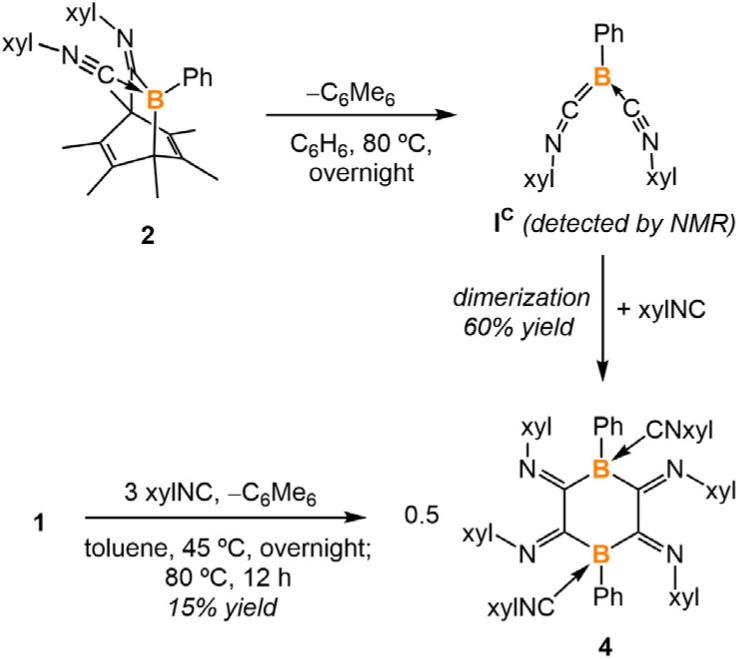
Synthesis of the tetraiminyl-1,4-diboracyclohexane 4.

It is hypothesized that a retro-Diels–Alder reaction occurred on 2 at the elevated temperature, producing hexamethylbenzene and boraketenimine (xylNC)_2_BPh as an intermediate (Scheme 2). Indeed, monitoring the reaction by ^11^B NMR spectroscopy revealed an intermediate at ^11B^*δ* = −17.0 ppm (Fig. S30†), comparable to the bulker boraketenimine TpB(CNMe)_2_ (Tp = 2,4,6-triisopropylphenyl) reported by Braunschweig (^11B^*δ* = −18.8 ppm).^6*a*^ Moreover, compound 4 was prepared *via* the reaction of compound 1 with xylNC (3 equiv.). However, the one-pot reaction results in significantly lower yields of 4 (15%) compared to the two-step synthesis (60%).

**Fig. 4:**
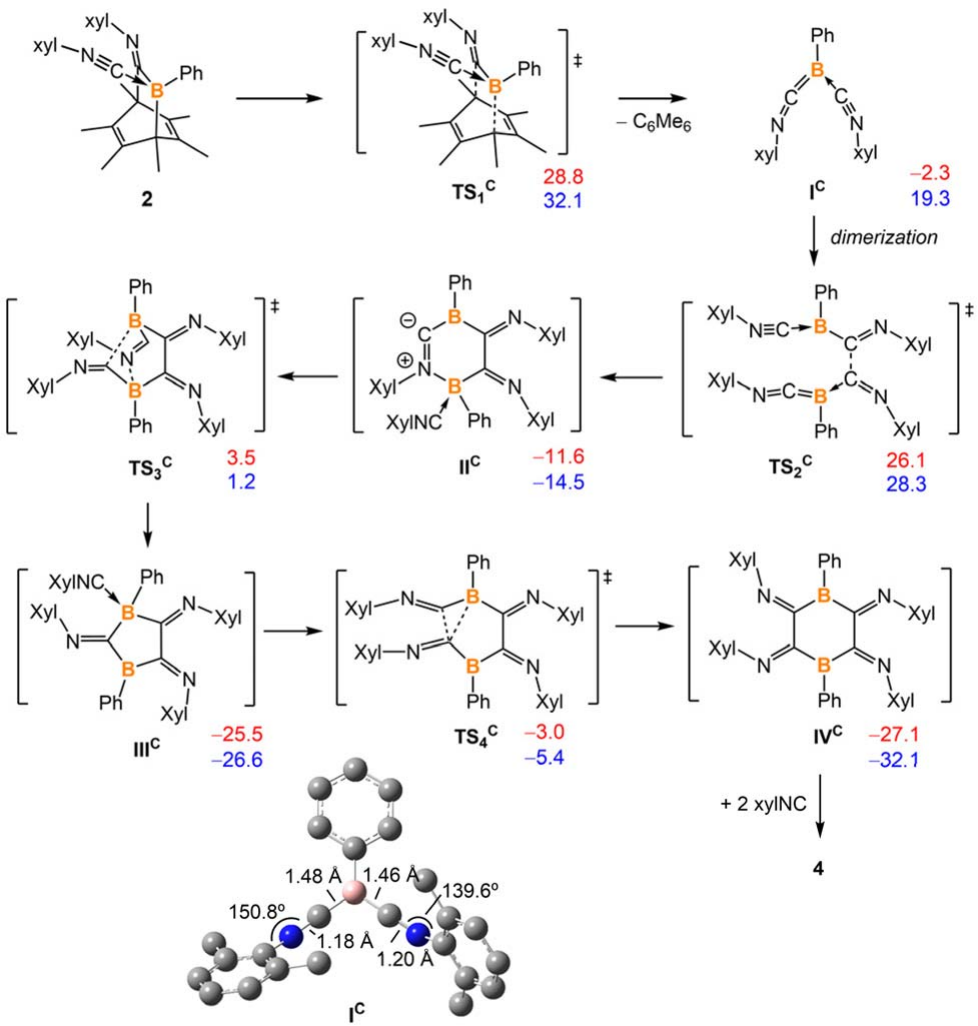
Energy profiles were calculated for the reaction of 2 to 4. The relative Gibbs free energies (in red) and electronic energies (in blue) are calculated at 298 K and given in kcal mol^−1^. The calculation is at M06-2X-D3/6-311g** level of theory with applied solvation models in toluene.

Density Functional Theory (DFT) calculations indicate that the energy barrier of this retro-Diels–Alder reaction is 28.8 kcal mol^−1^, which is surmountable at the given temperature. As indicated by the different N–C–N angles in the optimized structure (Fig. 4, bottom), the intermediate I^C^ is best described as a boraketenimine stabilized by coordination with a neutral isocyanide. Though the following steps, which finally led to the formation of 4, are experimentally elusive, DFT computations suggest a possible pathway comprising C–C coupling, ring contraction, and ring expansion (Fig. 4). The barrier of the C–C coupling process is computed to be 28.4 kcal mol^−1^, comparable to that of the retro-Diels–Alder step. The subsequent ring contraction and ring expansion represent the formal coupling of the other pair of carbons. We will show that a strong Lewis acid could abstract the coordinated isocyanide in 2. Upon formation of the dimerized product IV^C^, two Lewis acidic boron centers could abstract two isocyanides from 2, resulting in the isolated product 4. Overall, the Gibbs free energy of the dimerized product IV^C^ is 24.8 kcal mol^−1^ lower than its monomer I^C^.

**Scheme 3:**
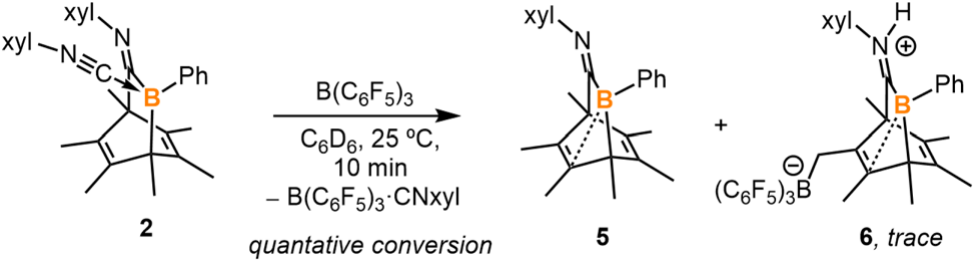
Abstraction of isocyanide ligand by a Lewis acid to generate 5.

**Fig. 5:**
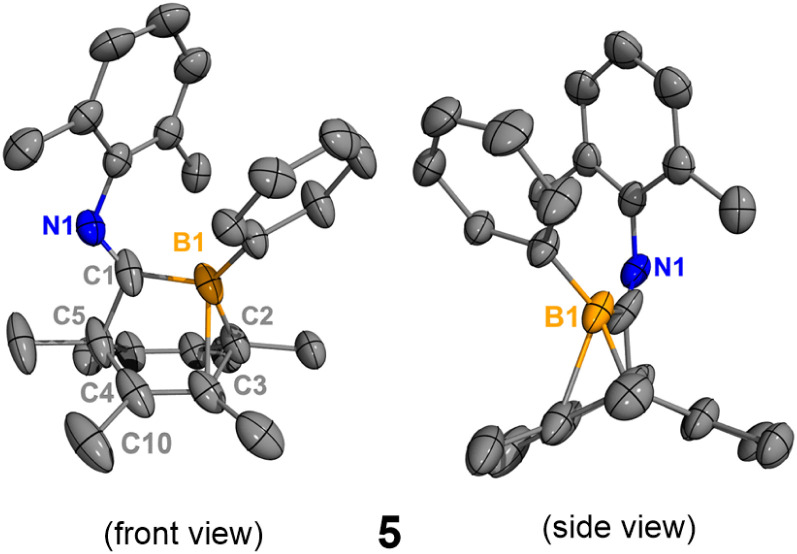
Molecular structure of 5. Hydrogen atoms have been omitted for clarity. The molecular structure of 6 is in the ESI (Fig. S32†). Thermal ellipsoids are drawn at the 50% probability level. Selected bond lengths [Å] and angles [°]: B1–C1 1.584(3), B1–C2 1.578(3), B1–C3 2.057(3), C2–C3 1.544(3), C3–C4 1.358(3), C4–C5 1.529(4), C4–C10 1.503(3), C1–B1–C2 109.6(1), C1–B1–C3 95.2(1), B1–C2–C3 82.4(1), C5–C1–B1–C2 31.4(2).

In order to obtain the uncoordinated bora-bicyclo[2.2.2]octa-2,5-diene, aiming to generate a base-free boraketenimine, a strong Lewis acid was used to abstract the isocyanide from the boron center (Scheme 3). As indicated by ^11^B NMR spectroscopy, the treatment of 2 with equimolar B(C_6_F_5_)_3_ resulted in the clean formation of B(C_6_F_5_)_3_·xylNC (^11B^*δ*: −20.9 ppm) and the uncoordinated bora-bicyclo[2.2.2]octa-2,5-diene 5 (^11B^*δ*: 41.7 ppm). While pure 5 does not crystallize on its own, storing a concentrated hexanes solution of the reaction mixture at −35 °C for 15 days gave a few yellow crystals, determined to be 5 and 6 by SC-XRD analyses. Each unit cell contains one molecule of 5 and one molecule of 6 (Fig. S32†). The B1–C1 (1.584(3) Å) and B1–C2 (1.573(3) Å) distances in 5 are remarkably shorter than those in 2 and 3 (Fig. 5). Interestingly, the B1–C3 distance in 5 is 2.057(3) Å, indicating a weak interaction between the electrophilic B1 and C3=C4 double bond. This C=C double bond is slightly longer than those in 2 and 3 (5, C3=C4 1.358(3) Å; 2, C4=C5 1.334(2) Å; 3, C5=C6 1.329(3) Å), and the boron atom in 5 is more distorted away from the C1–C2–C5 plane (C5–C1–B1–C2 31.4°). Compound 6 is not detectable by NMR spectroscopy due to its insufficient amount in the reaction mixture, and its formation may result from the C–H activation of 5. In addition, the isocyanide adduct 2 was regenerated from the treatment of 5 with equimolar xylNC. Therefore, compounds 2 and 5 are interconvertible by treating them with the respective Lewis acid or base.

**Scheme 4:**
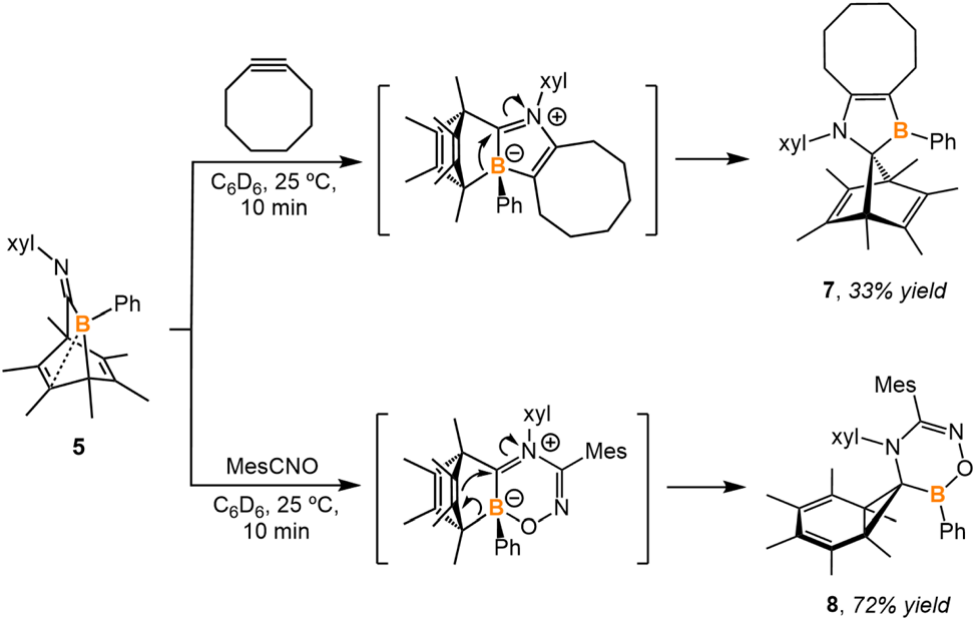
Treatment of compound 5 with cyclooctyne and mesityl nitrile *N*-oxide.

Compound 5 does not undergo retro-Diels–Alder reaction to release a base-free boraketenimine (PhB = C=Nxyl) at elevated temperatures; instead, it decomposes into multiple unidentifiable species. To test the ability of compound 5 to act as a synthon for the base-free boraketenimine, 5 was treated with cyclooctyne and mesityl nitrile *N*-oxide (Scheme 4). Based on ^1^H NMR spectroscopy, the reaction of both reagents did not lead to the elimination of hexamethylbenzene. In both reactions, compound 5 reacts as 1,3-dipole, with boron representing the electrophilic site and nitrogen the nucleophilic site. The 1,3-dipole undergoes (3 + 2) cycloaddition with cyclooctyne and (3 + 3) cycloaddition with mesityl nitrile *N*-oxide. The following 1,2-migration of the 5-membered ring and 3,2-migration of the 6-membered ring afford a norbornadiene derivative 7 and a norcaradiene derivative 8, respectively. The solid-state structures of 7 and 8 were obtained *via* SC-XRD analysis (Fig. S34 and S35†).

### Generating a transient oxoborane

Following the study of the reactivity with isocyanide, we anticipated that the reaction of 1 with trimethylamine *N*-oxide (TMAO) would afford a precursor to elusive oxoborane species. Currently, there are only a handful of examples for generating transient oxoborane, and a free oxoborane (RB≡O) has not been isolated.^17^

**Scheme 5:**
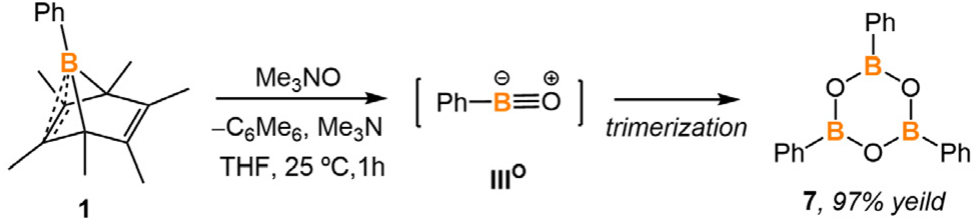
Generation of phenyl oxoborane intermediate and its trimerization.

In contrast to the reaction of 1 with isocyanide, the reaction between 1 and TMAO in THF was very fast, and both starting materials were consumed within an hour at room temperature (Scheme 5). The ^1^H NMR spectrum of the reaction mixture displayed a singlet at 2.13 ppm in C_6_D_6_, indicating the formation of hexamethylbenzene. After removing hexamethylbenzene from the reaction mixture by sublimation, the remaining solids were analyzed by ^11^B NMR spectroscopy in CDCl_3_, showing a singlet at 29.0 ppm. The ^1^H NMR spectrum is in accordance with triphenylboroxine (9) in CDCl_3_. It is known that the formation of 9 results from the trimerization of oxoboranes.^18^ However, our attempts to trap the oxoborane intermediate with other reagents were unsuccessful (Fig. S31†).

**Fig. 6:**
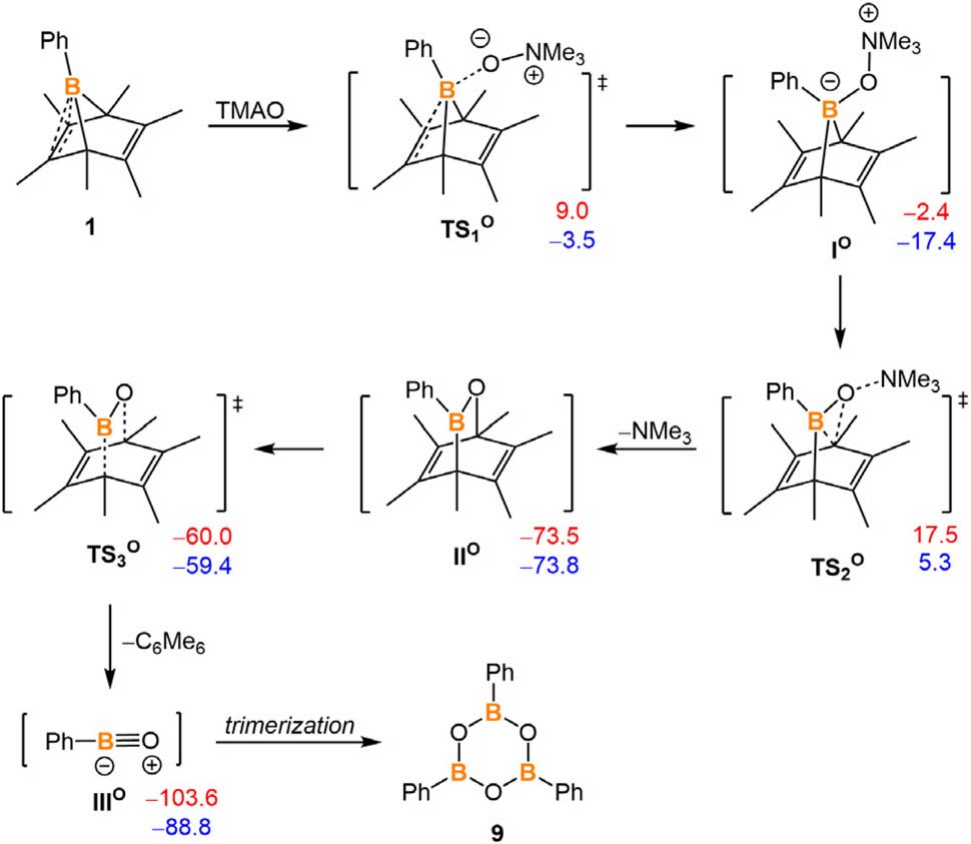
Proposed intermediates and transition states for the reaction of 1 to 9. The relative Gibbs free energies (in red) and electronic energies (in blue) are calculated at 298 K and given in kcal mol^−1^. The calculation is at M06-2X-D3/6-311g** level of theory with applied solvation models in toluene.

DFT calculations suggest that compound 1 and TMAO could first form a Lewis adduct I^O^. The geometry of I^O^ is pre-organized for oxygen insertion and trimethylamine leaving (Fig. S37†). This oxygen insertion step is very exergonic (−73.5 kcal mol^−1^), and its energy barrier for accessing the intermediate II^O^ is only 19.9 kcal mol^−1^. The release of oxoborane from hexamethylbenzene constitutes a retro-Diels–Alder reaction *via*TS_3_^O^. Surprisingly, the energy barrier of this retro-Diels–Alder reaction is remarkably low (13.5 kcal mol^−1^) compared to that of the boraketenimine leaving step (28.8 kcal mol^−1^), indicating that the formation of oxoborane is very favorable (Fig. 6).

### Generating a transient iminoborane

**Scheme 6:**
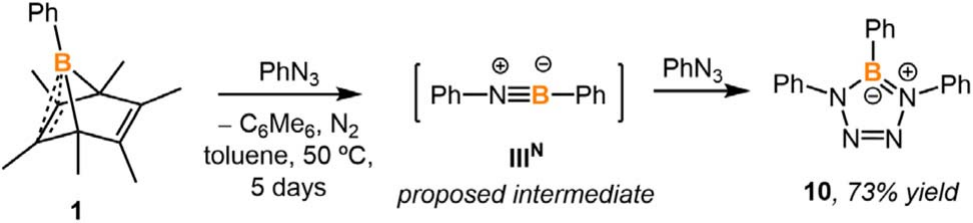
Generation of diphenyl iminoborane intermediate and its trapping with phenyl azide to afford compound 10.

Compared to boraketenimine and oxoborane, iminoborane is more well-studied.^19^ Transient iminoborane is commonly generated *via* N_2_ loss from the corresponding azidoborane (R_2_BN_3_) precursors.^20^ To examine whether the hexamethylbenzene platform could generate transient iminoborane, compound 1 was treated with phenyl azide at 50 °C (Scheme 6). The ^1^H NMR spectrum of the reaction mixture indicated the quantitative generation of hexamethylbenzene. The boron-containing species was separated from the mixture by washing with hexanes and recrystallization. SC-XRD analysis displays a BN_4_ 5-membered ring 10, known from (3 + 2) cycloaddition between iminoborane PhB≡NPh and phenyl azide.^8*b*,21^ The center boron in 10 gives a resonance of 25.4 ppm in the ^11^B NMR spectrum (Fig. 8). The nucleus-independent chemical shift (NICS) values of compound 10 (NICS(0) = −7.1; NICS(1) = −7.2) indicate that it possesses a certain degree of aromaticity.^22^ It should be noted that no intermediates were observed by NMR spectroscopy during the formation of 10.

**Fig. 7:**
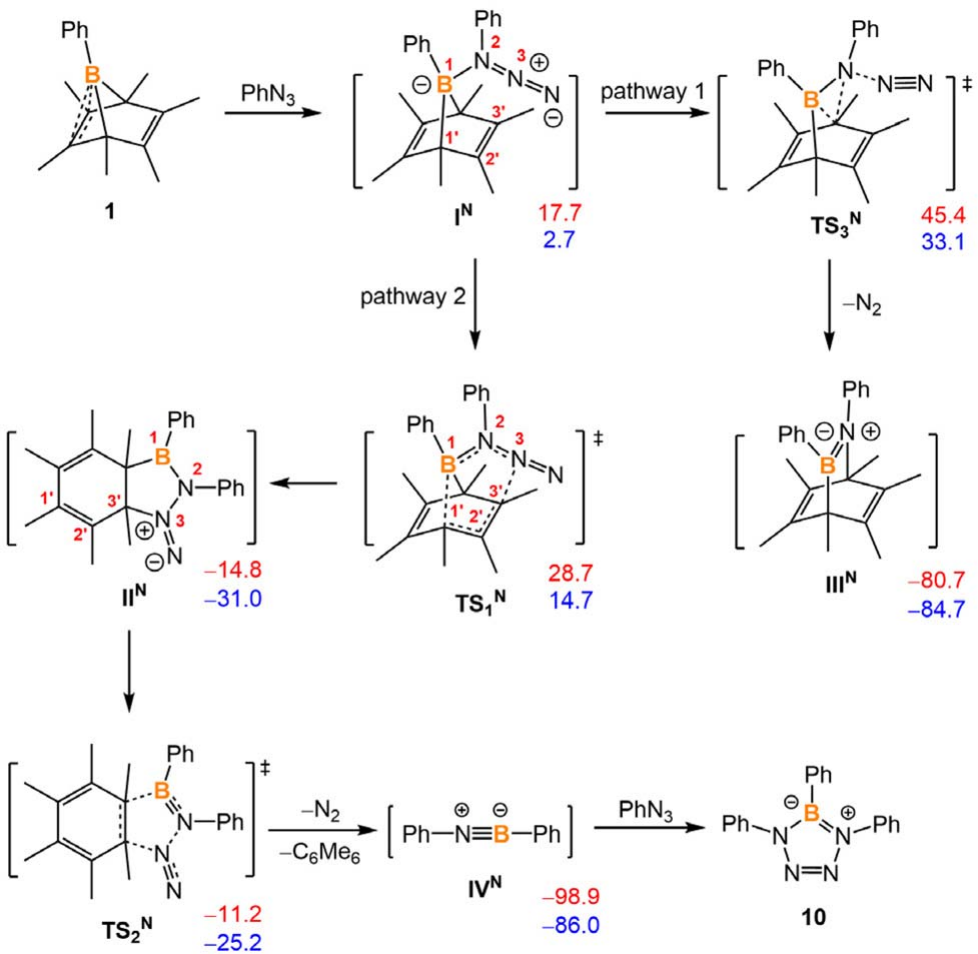
Proposed intermediates and transition states for the reaction from 1 to 10. The relative Gibbs free energies (in red) and electronic energies (in blue) are calculated at 298 K and given in kcal mol^−1^. The calculation is at M06-2X-D3/6-311g** level of theory and applied solvation models in toluene.

DFT calculations were performed to better understand the mechanism of iminoborane generation (Fig. 7). Compound 1 and phenyl azide could first form a Lewis acid-base adduct I^N^. Phenyl azide would act as a nitrene source to afford III^N^ as an intermediate, and the iminoborane evolves a retro-Diels–Alder reaction from III^N^, analogous to the formation of (xylNC)_2_BPh from 2. However, the overall barrier of this reaction pathway is too high to consider (45.4 kcal mol^−1^). Alternatively, the adduct I^N^ could undergo a [3,3]-sigmatropic rearrangement, leading to the formation of a bicyclic 6-fused-5 membered ring II^N^. This [3,3]-sigmatropic rearrangement is an extension of the Cope rearrangement (Fig. S36†). Intermediate II^N^ is extremely unstable and could subsequently undergo fragmentation to hexamethylbenzene, dinitrogen, and iminoborane by overcoming a very low barrier (TS_2_^N^, 3.6 kcal mol^−1^). The overall energy barrier for this alternative pathway is only 28.7 kcal mol^−1^, which is surmountable at the given temperature.

**Fig. 8:**
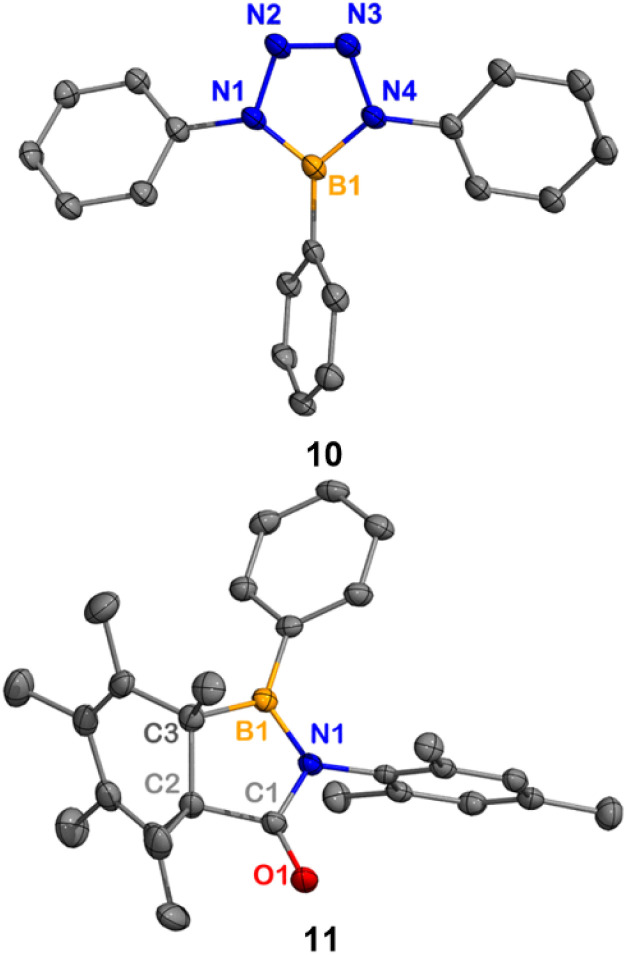
Molecular structures of 10 and 11 in the solid-state. Hydrogen atoms have been omitted for clarity. Thermal ellipsoids are drawn at the 50% probability level. Selected bond lengths [Å] and angles [°]: for 10, B1–N1 1.432(2), B1–N4 1.425(2), N1–N2 1.382(2), N2–N3 1.276(2), N3–N4 1.387(2), B1–N1–N2 109.9(1), N1–N2–N3 109.9(1), N1–B1–N4 100.7(1); for 11, B1–N1 1.423(2), N1–C1 1.401(1), C1–O1 1.211(2), C1–C2 1.533(2), B1–N1–C1 112.1(1), N1–C1–O1 124.1(1).

**Scheme 7:**
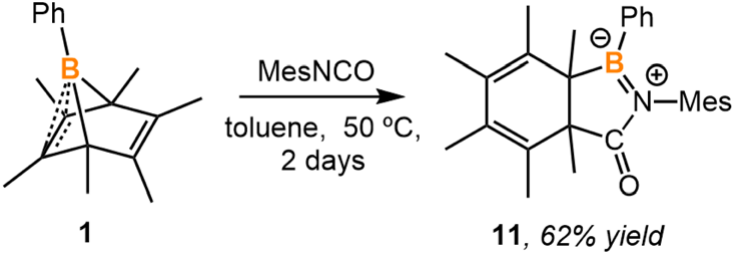
Synthesis of compounds 11.

In order to assess the feasibility of the second reaction pathway, compound 1 was treated with mesityl isocyanate, which is isoelectronic to phenyl azide. We proposed that treating 1 with isocyanate resulted in an analogous reaction to that of phenyl azide, while the bicyclic analog could not undergo hexamethylbenzene fragmentation at room temperature. Indeed, the reaction of 1 with mesityl isocyanate led to the expected 2,3-insertion product, affording bicyclic 11 (Scheme 7 and Fig. 8). Heating 11 at 50 °C showed no obvious fragmentation to hexamethylbenzene, iminoborane, and carbon monoxide.

## Conclusion

In conclusion, we have demonstrated that hexamethylbenzene elimination is a new synthetic method for constructing transient sterically unhindered boron-containing unsaturated bonds. The 1,1-insertion of isocyanide and an oxygen atom into boranorbornadiene results in boron-doped bicyclo[2.2.2]octa-2,5-dienes, which readily undergo retro-Diels–Alder reactions to afford the corresponding boraketenimine and oxoborane as intermediates. The 2,3-insertion of mesityl isocyanate and phenyl azide is reminiscent of a boron-involved Cope rearrangement. The fragmentation of the phenyl azide insertion product yields iminoborane, a species that readily undergoes (3 + 2) cycloaddition with phenyl azide. These reactions are interesting from a fundamental synthetic chemistry perspective, and the benzene extrusion strategy is helpful for the generation of novel boron-containing unsaturated species.

## Data availability

Experimental details, characterization, and computational details, including Fig. S1–S37 and Tables S1–S13,† X-ray crystallographic data for 2, 3, 4, 5, 6, 7, 8, 10, and 11 (CIF). CCDC identification codes 2299088–2299093, 2416228 and 2416229 are associated with the supplementary crystallographic data for this paper. These data can be obtained free of charge *via*http://www.ccdc.cam.ac.uk/data_request/cif.

## Author contributions

C. Z. conceptualized the project, synthesized and characterized the compounds, wrote the draft together with. P. D. and B. Y. E. T. R. J. G. and C. C. C. supervised the project, acquired financial support for the project, and revised the manuscript.

## Conflicts of interest

There are no conflicts to declare.

## Supplementary Material

SC-OLF-D5SC02645H-s001

SC-OLF-D5SC02645H-s002

SC-OLF-D5SC02645H-s003
