# Supplementary material for: Hexamethylbenzene elimination enables the generation of transient, sterically unhindered multiply bonded boron species
Source: Chem Sci. 2025 May 19;16(26):11841–8. doi: 10.1039/d5sc02645h (PMC12135023; doi:10.1039/d5sc02645h)
Supplement: SC-016-D5SC02645H-s001 [file SC-016-D5SC02645H-s001.pdf]

Supplementary Information (SI) for Chemical Science.
This journal is © The Royal Society of Chemistry 2025

# Hexamethylbenzene Elimination Enables the Generation of Transient, Sterically Unhindered Multiply Bonded Boron Species

Chonghe Zhang, Philipp Dabringhaus, Bi Youan E. Tra, Robert J. Gilliard, Jr* and Christopher C. Cummins*

## Table of Contents

**Synthetic details and characterization data** S2

General considerations S2

Synthesis of **2-11** S3-S24

Concentration dependence study of the reaction of **1** with xylNC S25

Monitoring the fragmentation of **2** S26

**Other attempted experiments** S28

**Crystal structures** S29

**Computational Studies** S37

**References** S40

## Synthetic details and characterization data

**General considerations**:

All air- and moisture-sensitive reactions were carried out under an inert atmosphere of argon using standard Schlenk techniques or in an MBRAUN LABmaster glovebox equipped with a –35 °C freezer. All glassware used for reactions were oven-dried overnight at 190 °C. Reaction solvents including toluene and hexanes were purified by distillation from Na/benzophenone. Deuterated solvents were purchased from Cambridge Isotope Laboratories and distilled from Na/benzophenone ($C_6D_6$).

$PhB(C_6Me_6)$,[1] cyclooctyne,[2] and mesitylnitrile oxide[3] were prepared according to literature procedures. $PhN_3$ (0.1 M in methyl *tert*-butyl ether) was purchased from Millipore-Sigma and degassed by the freeze-pump-thaw method and stored over activated 4 Å molecular sieves for 48 h in the glovebox prior to use. Unless otherwise noted, all other chemicals were purchased commercially and used as received.

NMR spectra were obtained on Bruker Avance 400, Avance 401, Neo 402, Neo 500, and Neo 501 spectrometers. $^{1}H$ and $^{13}C$ NMR spectra were referenced to residual solvent peaks of the deuterated solvent (for $C_6D_6$, $^{1}H$ = 7.16 ppm, $^{13}C$ = 128.06 ppm; for $CDCl_3$, $^{1}H$ = 7.16 ppm, $^{13}C$ = 128.06 ppm). $^{11}B$ NMR spectra were referenced to an external standard, $BF_3{\cdot}Et_2O$ ($^{11}B$: δ = 0.00). Background suppression was applied for all $^{11}B$ NMR spectra. Abbreviations are as follows; s = singlet, d = doublet, t = triplet, sept = septet, dt = doublet of triplets, m = multiplet, br = broad. Unless noted, all spectra were acquired at 25 ºC.

HRMS were obtained on high-resolution JEOL AccuTOF 4G LC-plus equipped with an ionSense DART (Direct Analysis in Real Time) source. Elemental analyses were obtained on Thermo Scientific™ FlashSmart™ Elemental Analyzers. IR was performed on an ALPHA II compact FT-IR spectrometer. Samples were removed from the glovebox in sealed vials and briefly handled in the air prior to data collection. Details for single-crystal diffraction measurements are given in the crystallography section of this document.

**Synthesis of 2:** In a vial, PhB($C_6Me_6$) (100 mg, 0.40 mmol, 1.0 eq.) and 2,6-xylyl isocyanide (106 mg, 0.81 mmol, 2.0 eq.) were dissolved in toluene (20 mL). The solution was stirred at room temperature for three days, and the color gradually turned yellow. The solution was concentrated to approx. 0.5 mL and hexanes (2 mL) was added. The mixture was stored in the freezer (–35 $^{o}$C) for 2 days to give a yellow precipitate. The mother liquor was removed by pipette, and the remaining solid was dried under vacuum to afford the final product. The product contains 10 percent of compound **3** and was used without further purification (compound **2**: 166 mg, 0.32 mmol, 81%).

Repeating the same procedures except using 2 mL toluene led to an increase in the production of **3**. The yellow precipitate was recrystallized with hexane/toluene three times to finally afford a mixture (~10 mg) of **2** and **3** (54: 46). The mixture was used to characterize compound **3**.

**Compound 2:**

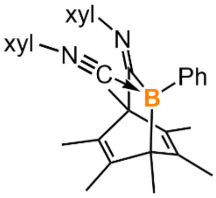


**$^{1}$H NMR** (500 MHz, $C_6D_6$): 1.43 (s, 3H), 1.49 (s, 3H), 1.80 (s, 6H), 1.86 (s, 3H), 1.89 (s, 3H), 1.95 (s, 6H), 2.05 (s, 3H), 2.06 (s, 3H), 6.19 (d, $J$ = 7.2 Hz, 1H), 6.46 to 6.52 (m, 3H), 6.65 (d, $J$ = 7.4 Hz, 1H), 6.73 (t, $J$ = 7.7 Hz, 1H), 7.10 to 7.20 (m, 5H). **$^{11}$B NMR** (161 MHz, $C_6D_6$): –17.82 (s). **$^{13}$C{$^{1}$H} NMR** (126 MHz, $C_6D_6$): 13.93 (s), 14.62 (s), 16.02 (s), 16.04 (s), 17.16 (s), 17.75 (s), 18.70 (s), 18.85 (s), 18.89 (s), 60.75 (s), 121.15 (s), 125.18 (s), 125.60 (s), 126.38 (s), 126.27 (s), 128.57 (s), 129.33 (s), 130.40 (s), 130.58 (s), 132.77 (s), 134.14 (s), 136.28 (s), 139.45 (s), 140.86 (s), 153.51 (s). **HRMS** (m/z): $[M+H]^+$ calcd. for $C_{36}H_{42}BN_2$, 513.34410, not found; calcd. for $C_{27}H_{33}BN$ (xylNC dissociates), 382.27060, found 382.2751.

**Compound 3:**

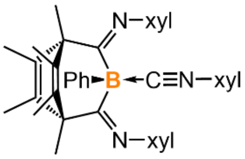


**$^{1}$H NMR** (500 MHz, $C_6D_6$): 1.70 (br. s, 6H), 1.76 (br. s, 6H), 1.81 (br. s, 6H), 1.96 (s, 6H), 1.98 (br. s, 6H), 6.42 (d, $J$ = 7.5 Hz, 2H), 6.53 to 6.60 (m, 5H). (Some peaks are hidden and thus unable to identify) **$^{11}$B NMR** (161 MHz, $C_6D_6$): –13.51 (s). **$^{13}$C{$^{1}$H} NMR** (126 MHz, $C_6D_6$): 16.51 (s), 17.26 (s), 18.40 (s), 19.81 (s), 21.23 (s), 21.43 (s), 23.06 (s), 31.97 (s), 58.96 (s), 121.29 (s), 125.70 (s), 125.75 (s), 126.70 (s), 127.52 (s), 130.32 (s), 134.05 (s), 134.92 (s), 135.76 (s). **HRMS** (m/z): $[M+H]^+$ calcd. for $C_{45}H_{51}BN_3$, 644.41760, not found; calcd. for $C_{36}H_{42}BN_2$ (xylNC dissociates), 513.34410, found 513.35032.

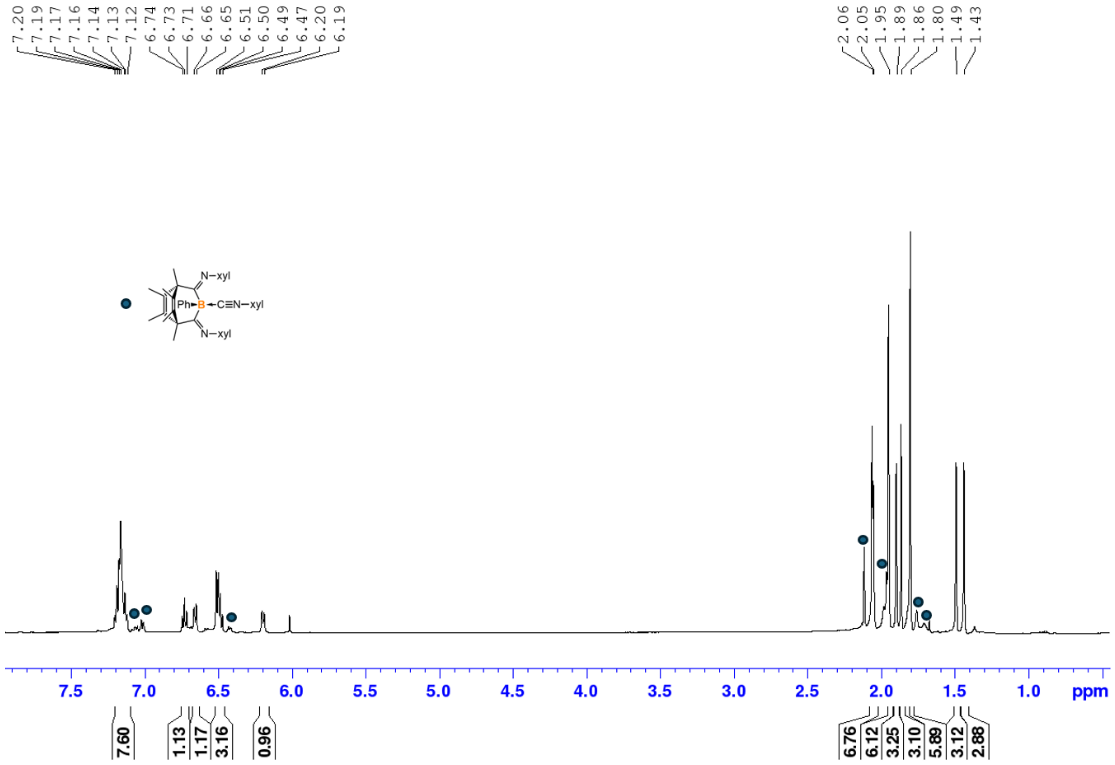


**Figure S1.** $^{1}H$ NMR spectrum of **2** in $C_6D_6$

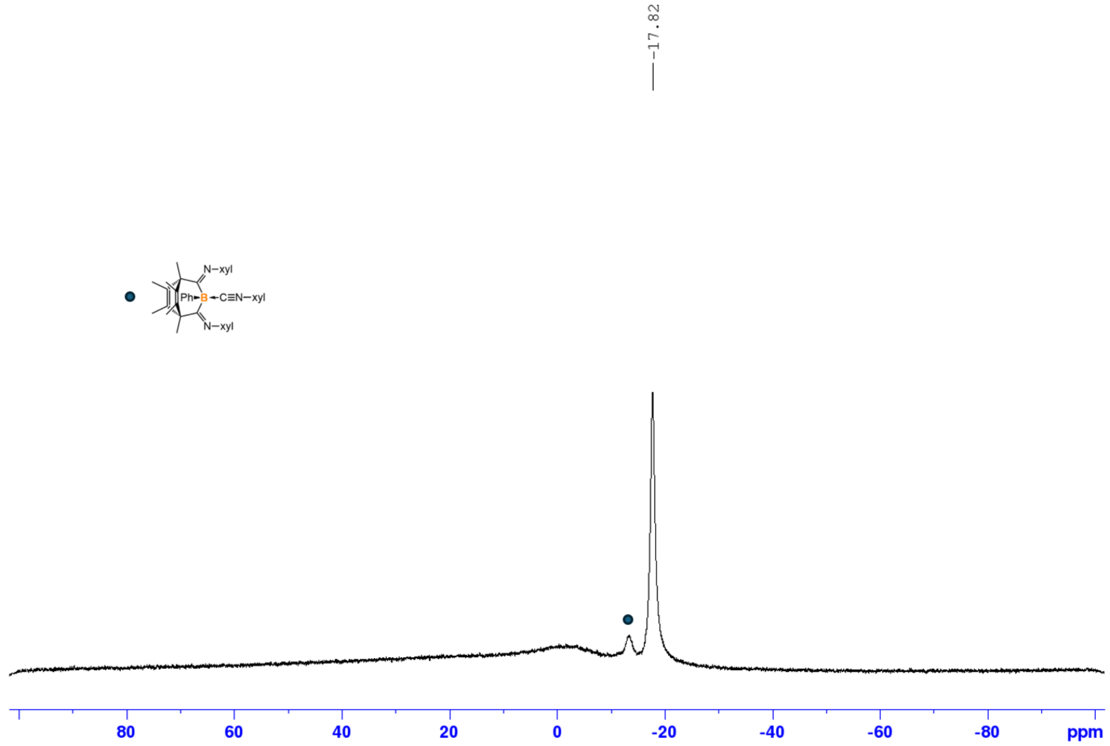


**Figure S2.** $^{11}B$ NMR spectrum of **2** in $C_6D_6$

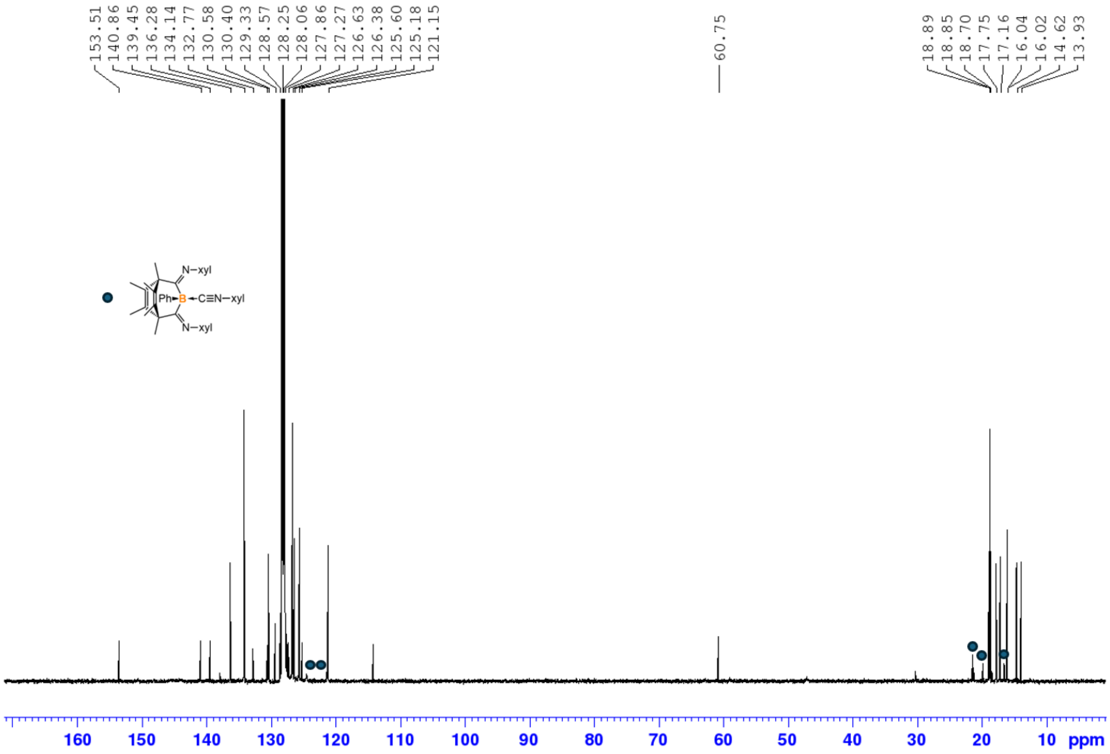


**Figure S3.** $^{13}C\{^1H\}$ NMR spectrum of **2** in $C_6D_6$

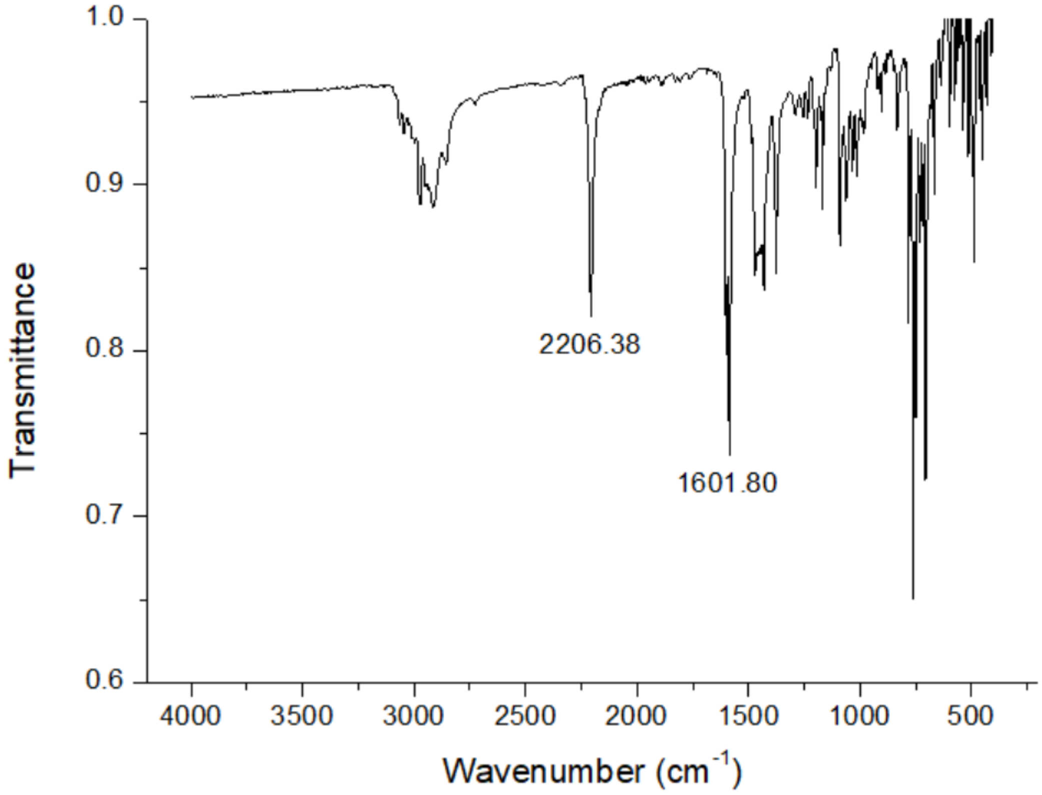


**Figure S4.** IR spectrum of **2**

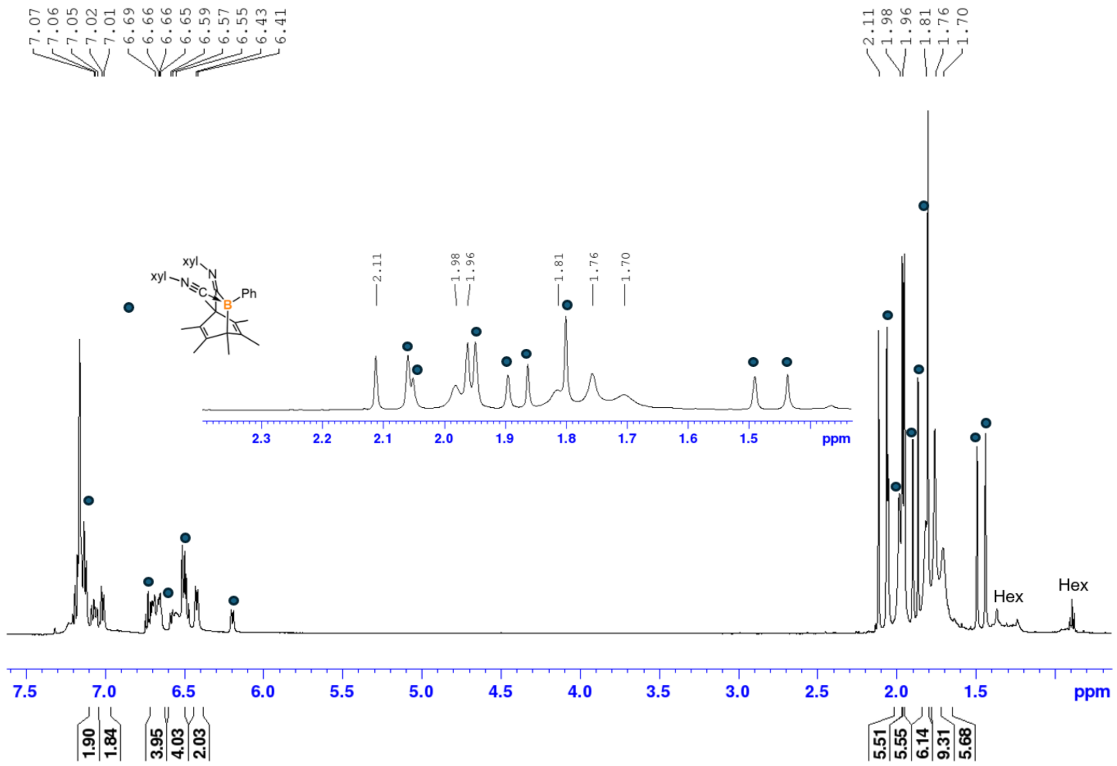


**Figure S5.** $^{1}H$ NMR spectrum of **3** in $C_6D_6$ (mixed with **2**, only the peaks belonging to **3** were picked and integrated)

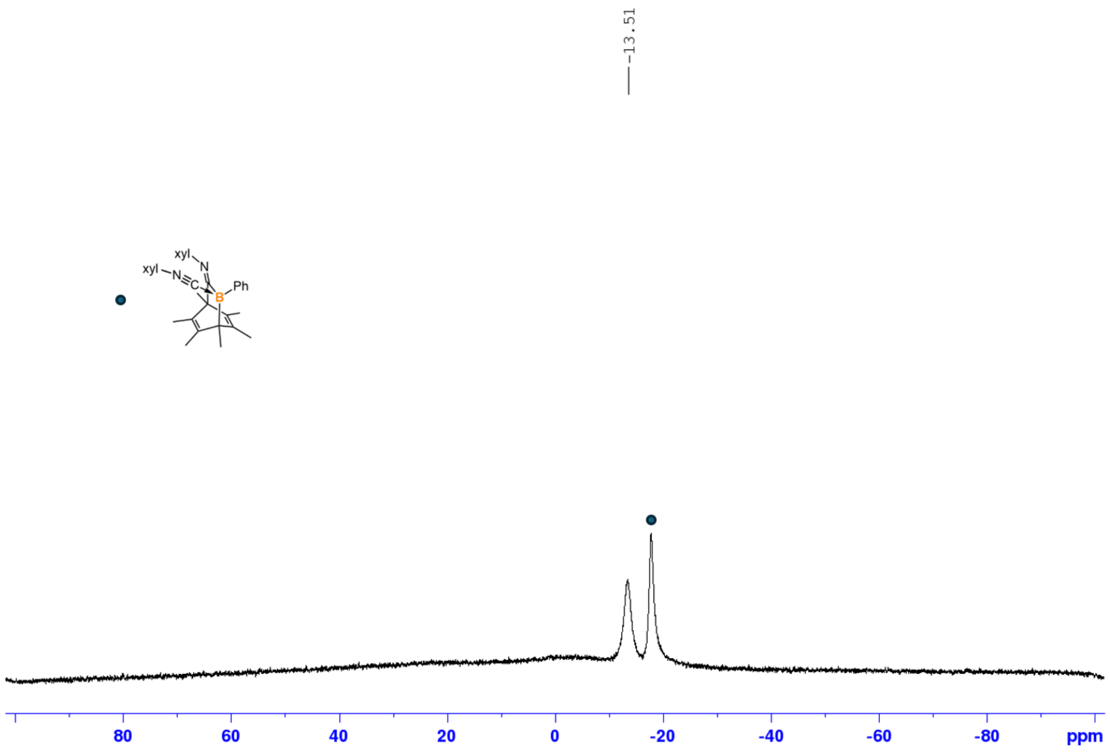


**Figure S6.** $^{11}B$ NMR spectrum of **3** in $C_6D_6$ (mixed with **2**, only the peaks belonging to **3** were picked)

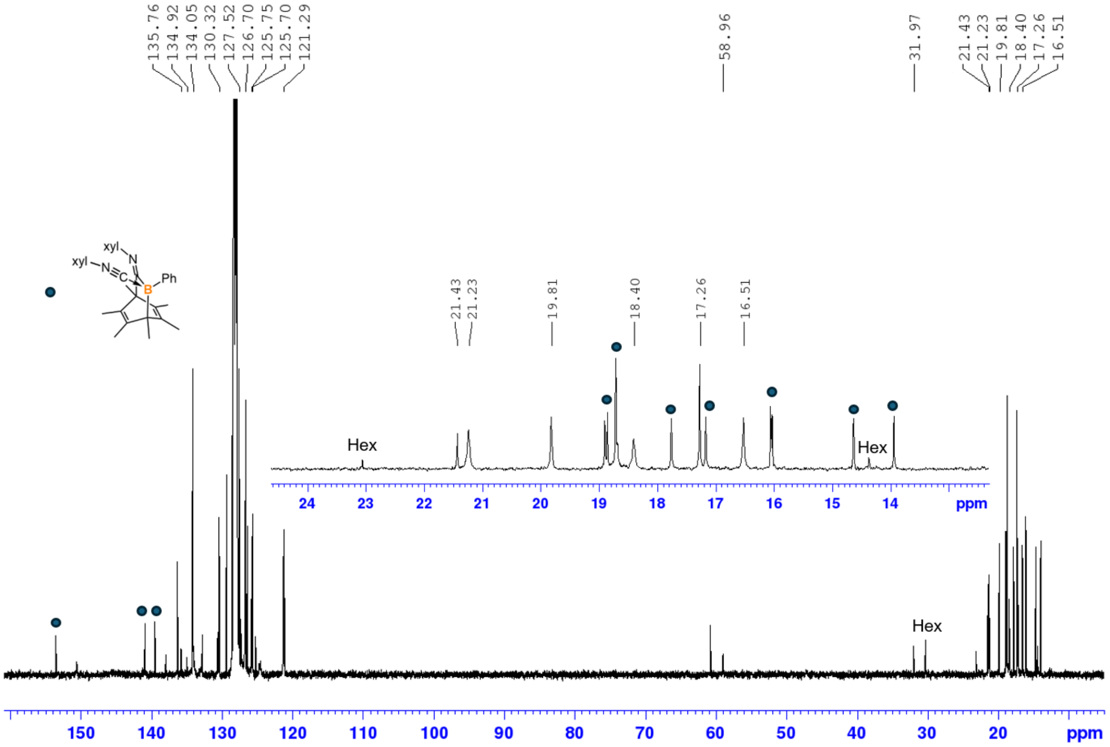


**Figure S7.** $^{13}C\{^1H\}$ NMR spectrum of **3** in $C_6D_6$ (mixed with **2**, only the peaks belonging to **3** were picked)

**Synthesis of 4:** In a Schlenk tube, compound **2** (75 mg, 0.15 mmol) was dissolved in benzene (1 mL). The solution was stirred and heated at 80 °C overnight, and the color gradually turned from yellow to deep dark red. The solution was slowly cooled down to room temperature and yellow crystalline solids were precipitated. The liquid was removed by filtration, and the solid was dried under vacuum to afford the final product (25 mg, 0.022 mmol, 60.3%). Compound **4** was insoluble in nearly all solvents (DCM, toluene, benzene, THF, acetone) but gave NMR signals at 105 °C in d8-toluene.

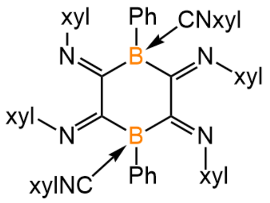


One pot synthesis: $PhB(C_6Me_6)$ (615 mg, 2.54 mmol, 1.0 eq.) and 2,6-dimethylphenyl isocyanide (1000 mg, 7.62 mmol, 3.1 eq.) were dissolved in 100 mL toluene. The solution was stirred and heated at 45 °C overnight and the color gradually turned dark yellow. The solution was concentrated to approx. 20 mL and heated at 80 °C for another 12 hours. The solution gradually turned from yellow to deep dark red and yellow crystalline solids were precipitated. The liquid was removed by filtration and the solid was dried under vacuum as the final product (189 mg, 0.196 mmol, 15.4%).

**$^{11}$B NMR** (161 MHz, d8-toluene, 105 °C): –10.91 (s). **HRMS** (m/z): not found. **IR** ($cm^{-1}$): 2245.95 (C≡N stretching), 1567.72 (C=N stretching). **Elemental analysis**: calcd. for $C_{66}H_{64}B_2N_6\cdot 2C_6H_6$, C, 83.71; H, 6.85; N, 7.51; found, C, 83.52; H, 6.85; N, 7.58.

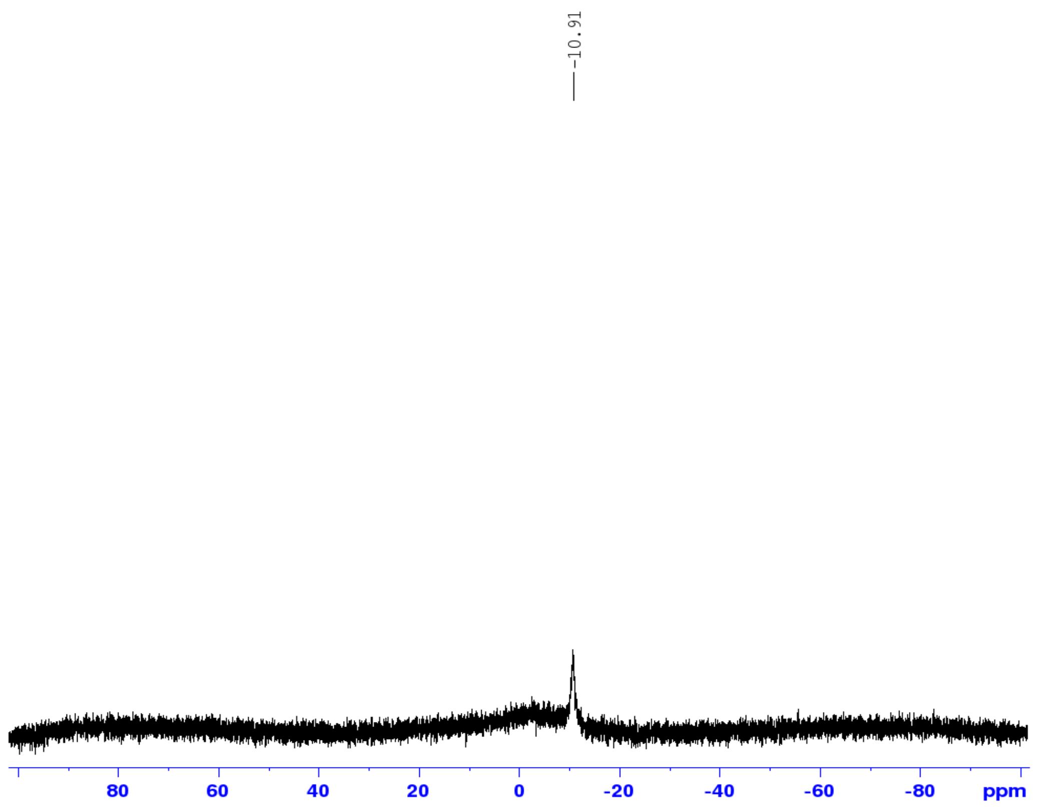


**Figure S8.** $^{11}$B NMR spectrum of **4** in d8-toluene

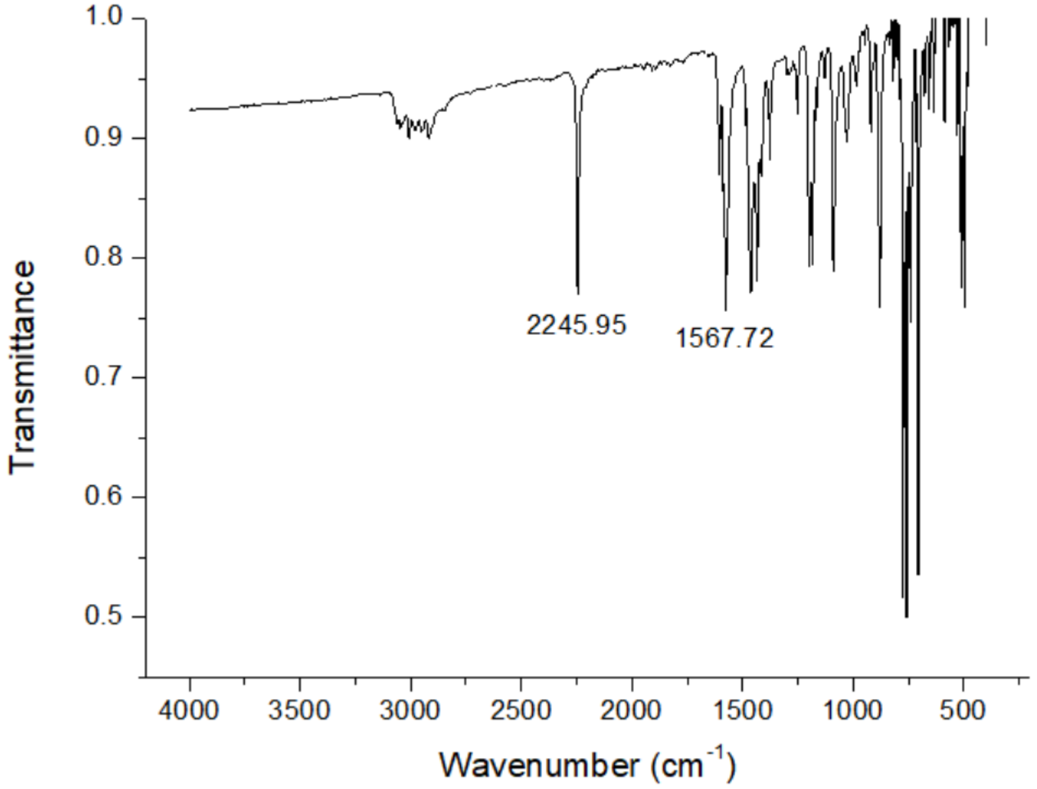


**Figure S9.** IR spectrum of **4.**

**Treatment of 2 with BCF**: In a vial, compound **2** (51 mg, 0.20 mmol, 1.0 eq.) and tris(pentafluorophenyl)borane (BCF) (51 mg, 0.20 mmol, 1.0 eq.) were dissolved in toluene (5 mL). The solution was stirred at room temperature for 10 min, and all volatile materials in the solution were removed under vacuum to afford a sticky solid. $^{1}H$ and $^{11}B$ NMR spectroscopies suggested the reaction led to a quantitative conversion to BCF· xylNC adduct and compound **5**. The sticky solid was redissolved into hexane (approx. 1 mL) and stored at –35 °C for a week. Partial BCF· xylNC adduct precipitated as a colorless crystalline solid. The mother liquor was transferred to a new vial and concentrated to approx. 0.5 mL. The solution was stored at –35 °C for another week, and a few yellow crystals precipitated, identified as compounds **5** and **6** by XRD analysis. Compound **5** was co-crystallized with compound **6** in one unit cell. BCF· xylNC was also synthesized by the reaction of BCF with xylNC.

BCF· xylNC: **$^{1}$H NMR** (400 MHz, $C_6D_6$): 1.81 (s, 6H), 6.36 (d, *J* = 7.6 Hz, 2H), 6.65 (t, *J* = 7.7 Hz, 1H). **$^{11}$B NMR** (128 MHz, $C_6D_6$): –20.92 (s). **$^{13}$C{$^{1}$H} NMR** (101 MHz, $C_6D_6$): 17.27 (s), 128,77 (s), 132.28 (s), 137.45 (s), 137.86 (m),141.02 (m), 148.55 (m). **$^{19}$F NMR** (376 MHz, $C_6D_6$): –162.57 (td, *J* = 23.6, 9.1 Hz), –154.97 (t, *J* = 22.0 Hz), –132.19 (dd, *J* = 24.0, 8.1 Hz).

Compound **5**: **$^{1}$H NMR** (500 MHz, $C_6D_6$): 1.39 (s, 3H), 1.50 (s, 6H), 1.67 (s, 3H), 1.75 (s, 6H), 1.98 (s, 6H), 6.63 (m, 3H), 6.76 (m, 2H), 6.91 (m, 3H). **$^{11}$B NMR** (161 MHz, $C_6D_6$): 41.82 (s). **$^{13}$C{$^{1}$H} NMR** (126 MHz, $C_6D_6$): 13.76 (s), 14.41 (s), 14.57 (s), 15.77 (s), 18.27 (s), 55.32 (s), 114.17 (s), 123.03 (s), 125.69 (s), 126.87 (s), 127.00 (s), 127.69 (s), 129.33 (s), 131.61 (s), 141.08 (s). **HRMS** (m/z): $[M+H]^+$ calcd. for $C_{27}H_{33}BN$, 382.27060, found 382.2751.

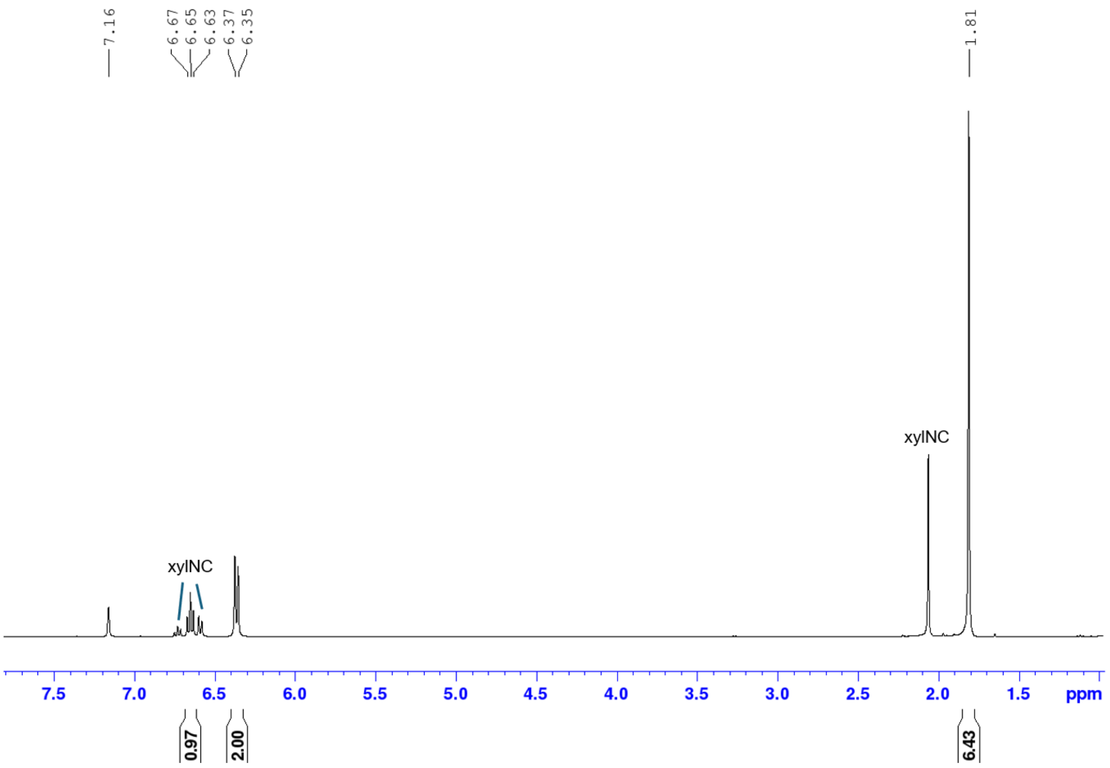


**Figure S10.** $^{1}H$ NMR spectrum of BCF· xylNC in $C_6D_6$

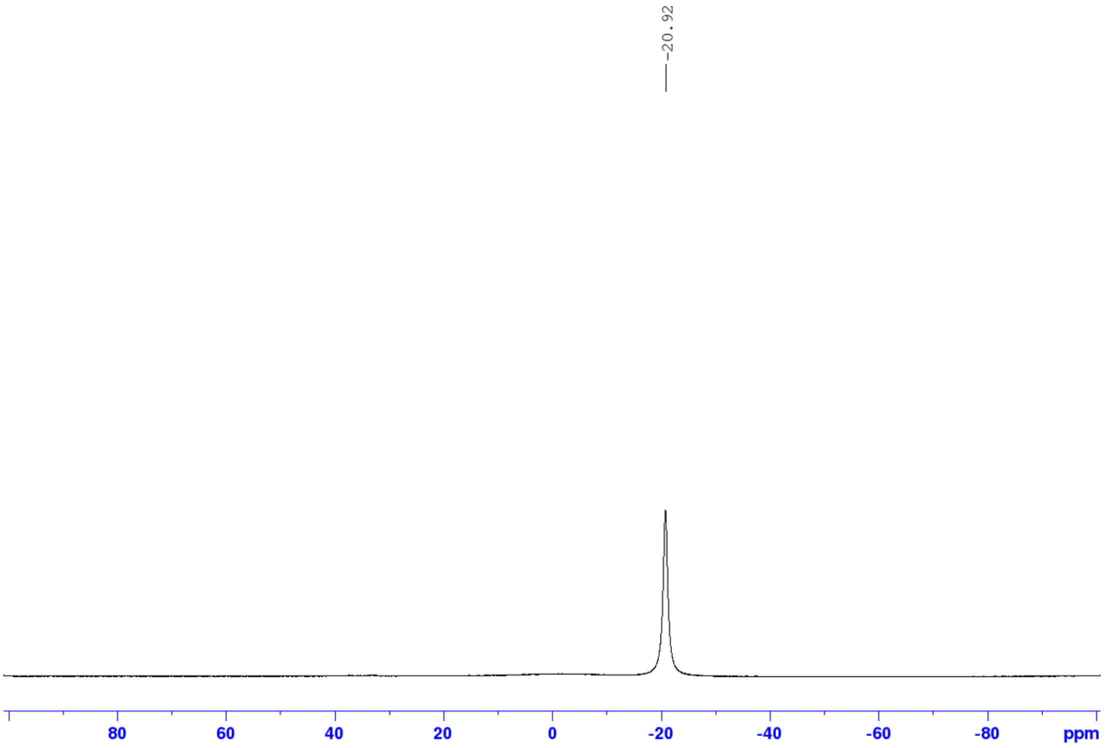


**Figure S11.** $^{11}B$ NMR spectrum of BCF· xylNC in $C_6D_6$

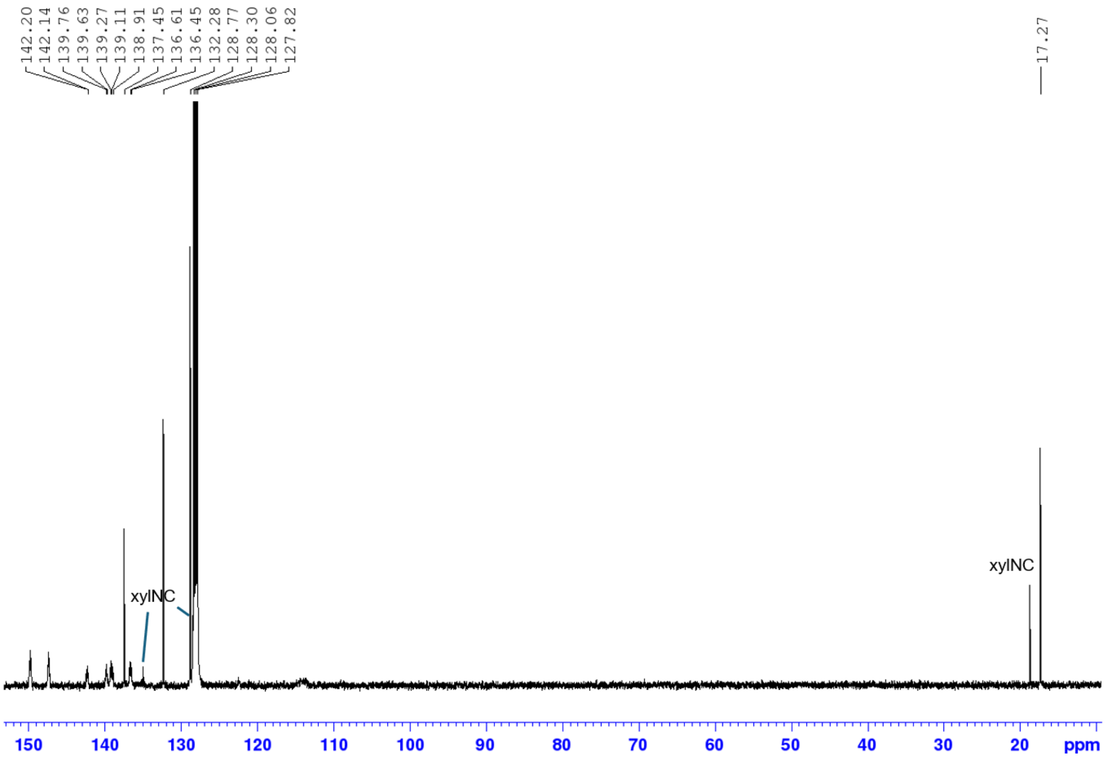


**Figure S12.** $^{13}C\{^{1}H\}$ NMR spectrum of BCF· xylNC in $C_6D_6$

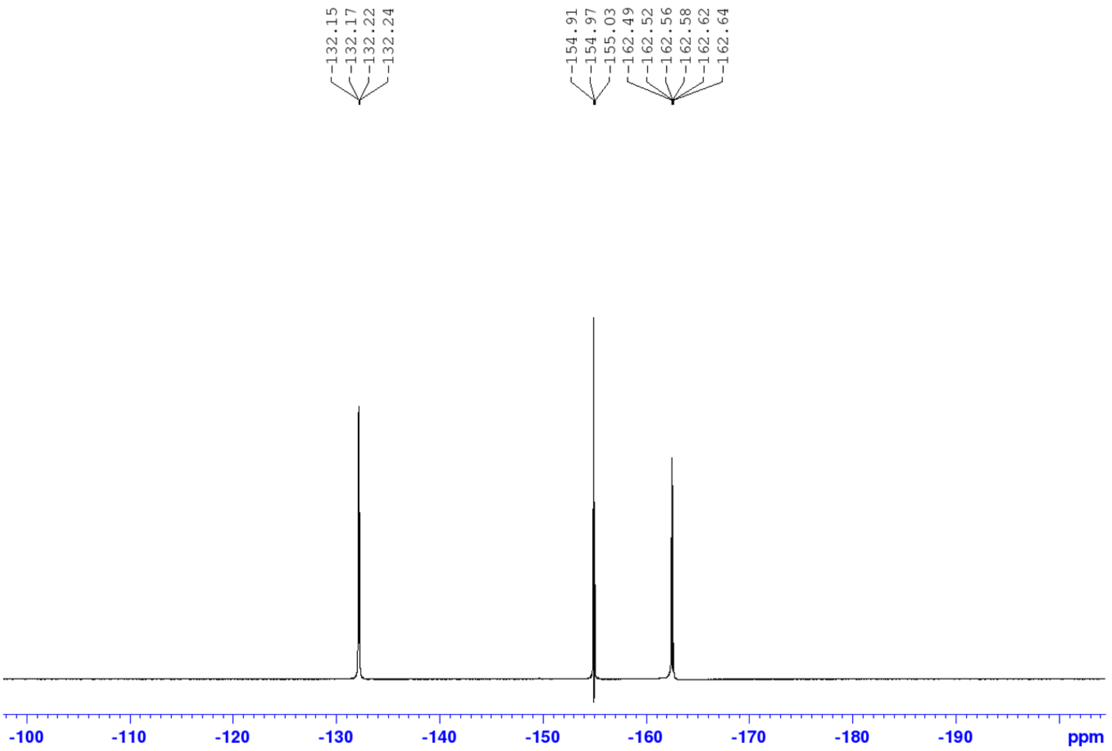


**Figure S13.** $^{19}F$ NMR spectrum of BCF· xylNC in $C_6D_6$

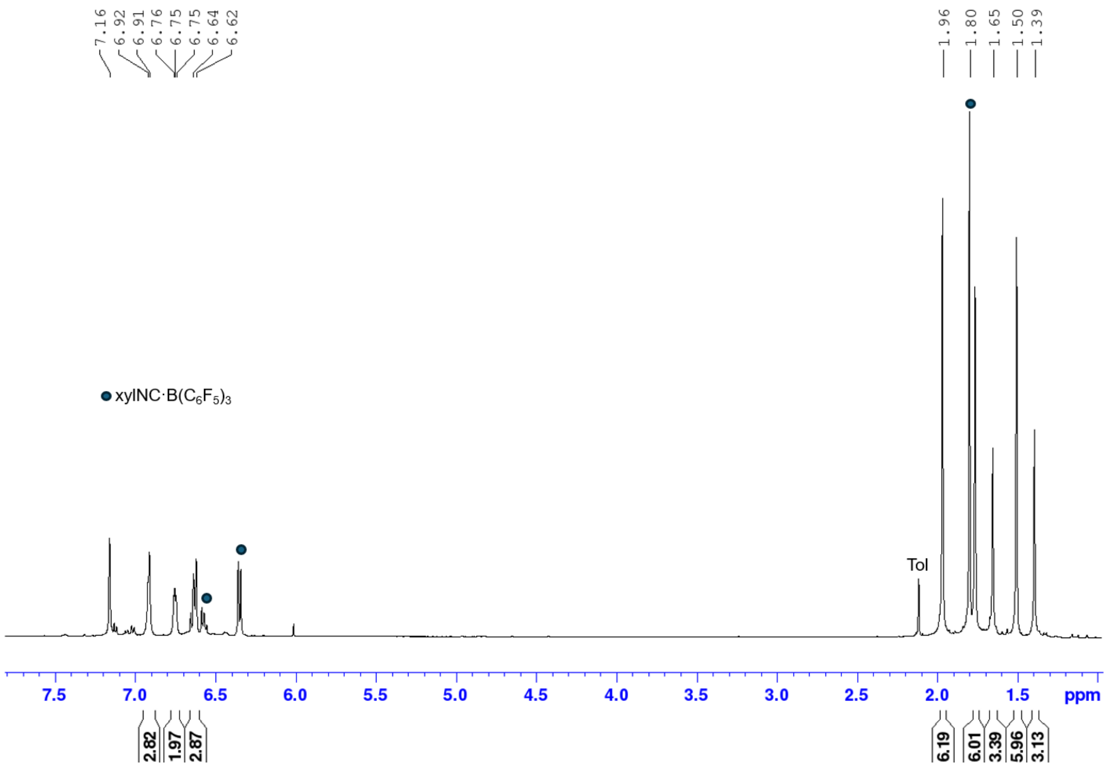


**Figure S14.** $^{1}H$ NMR spectrum of **5** in $C_6D_6$ (mixed with BCF· xylNC. Only the peaks belonging to **5** were picked and integrated)

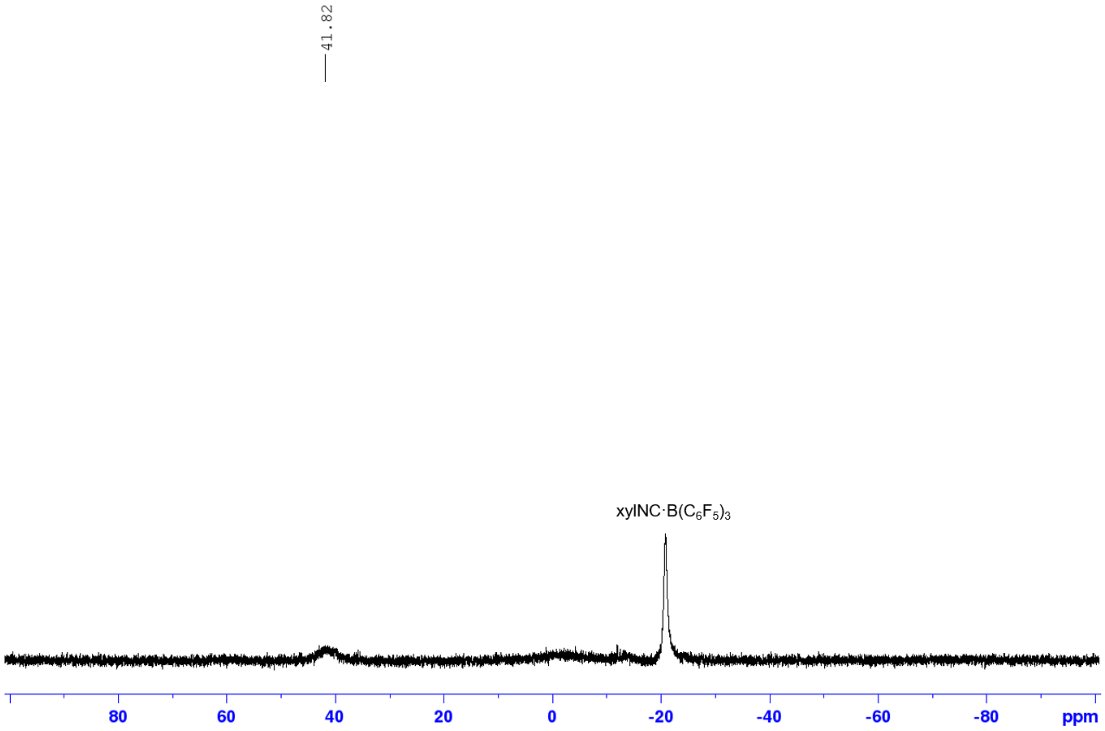


**Figure S15.** $^{11}B$ NMR spectrum of **5** in $C_6D_6$ (mixed with BCF· xylNC)

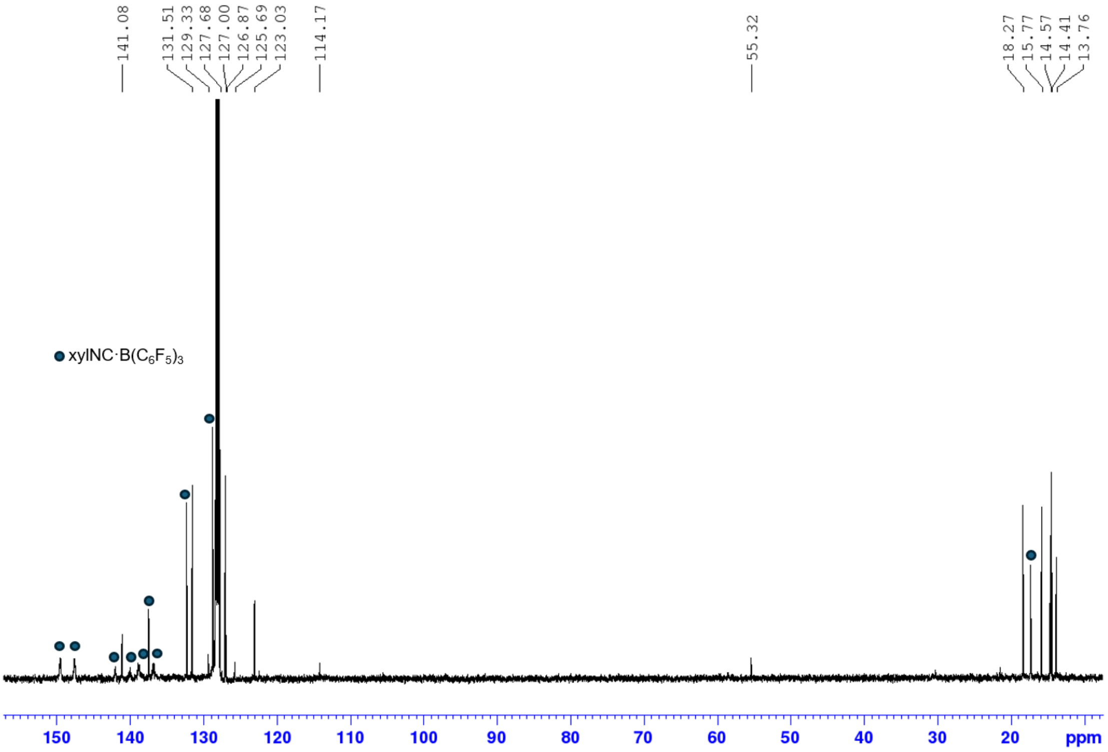


**Figure S16.** $^{13}C\{^{1}H\}$ NMR spectrum of **5** in $C_6D_6$ (mixed with BCF· xylNC)

## Synthesis of 7

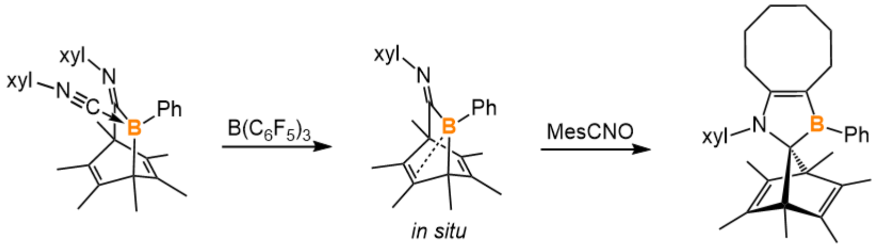


In a vial, compound **2** (217 mg, 0.42 mmol, 1.0 eq.) and $B(C_6F_5)_3$ (227.8 mg, 0.45 mmol, 1.05 eq.) were dissolved in 3 mL benzene. The solution was stirred for 5 min at 25 ºC. Cyclooctyne (48.7 mg, 0.45 mmol, 1.05 eq.) was added to the stirring solution. The mixture was stirred for 5 min. All volatile materials were removed by vacuum. Hexanes (3 mL) was added and the mixture was stirred for 30 min. The mixture was then filtered, and the filtration was concentrated and cooled in the freezer (–35 ºC) to afford the crystalline solid (72.6 mg, 0.15 mmol, 33.0%).

**$^1$H NMR** (500 MHz, $C_6D_6$): 1.25 (s, 6H), 1.26 (s, 6H), 1.31 (s, 6H), 1.43 (m, 2H), 1.56 (m, 2H), 1.67 (m, 2H), 2.02 to 2.06 (m, 8H), 2.32 (m, 2H), 6.85 (d, *J* = 7.5 Hz, 2H), 6.95 (t, *J* = 7.3 Hz, 1H), 7.17 (m, 1H), 7.30 to 7.36 (m, 4H). **$^{11}$B NMR** (161 MHz, $C_6D_6$): 64.55 (s). **$^{13}$C{$^1$H} NMR** (126 MHz, $C_6D_6$): 12.27 (s), 12.65 (s), 13.89 (s), 21.71 (s), 25.96 (s), 26.88 (s), 26.97 (s), 27.11 (s), 29.70 (s), 33.12 (s), 64.36 (s), 125.89 (s), 126.67 (s), 127.05 (s), 127.98 (s), 130.93 (s), 141.14 (s). **HRMS** (m/z): $[M+H]^+$ calcd. for $C_{35}H_{45}BN$, 490.36450; found: 490.36858.

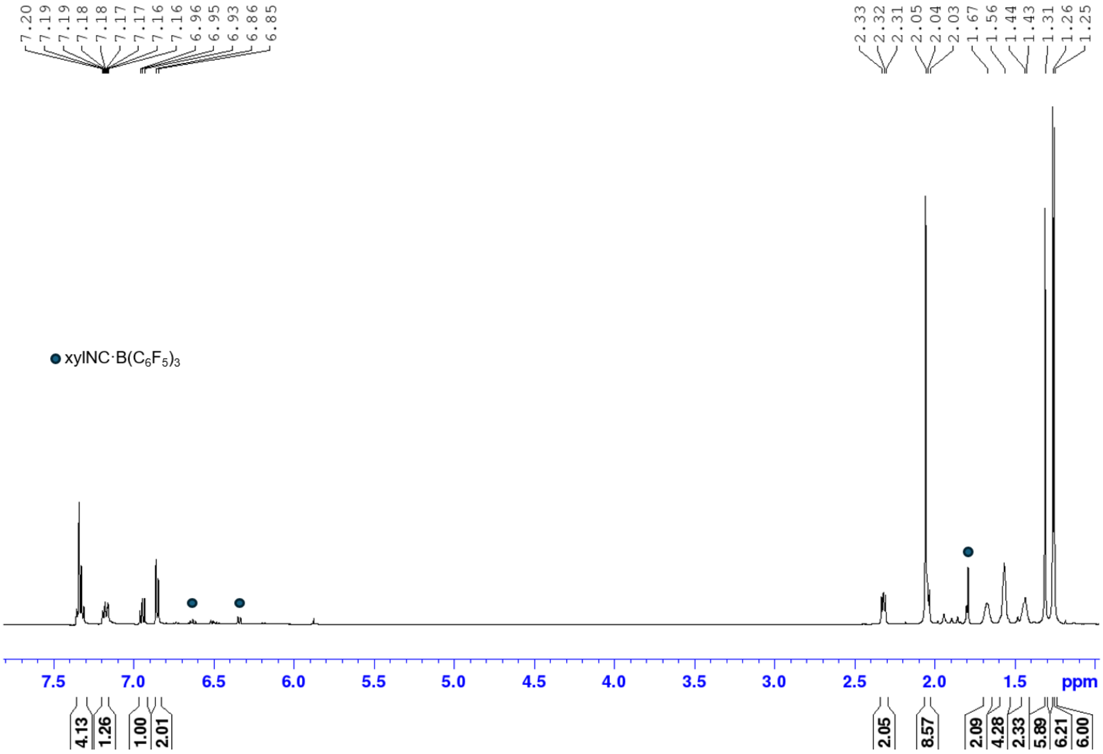


**Figure S17.** $^1$H NMR spectrum of **7** in $C_6D_6$

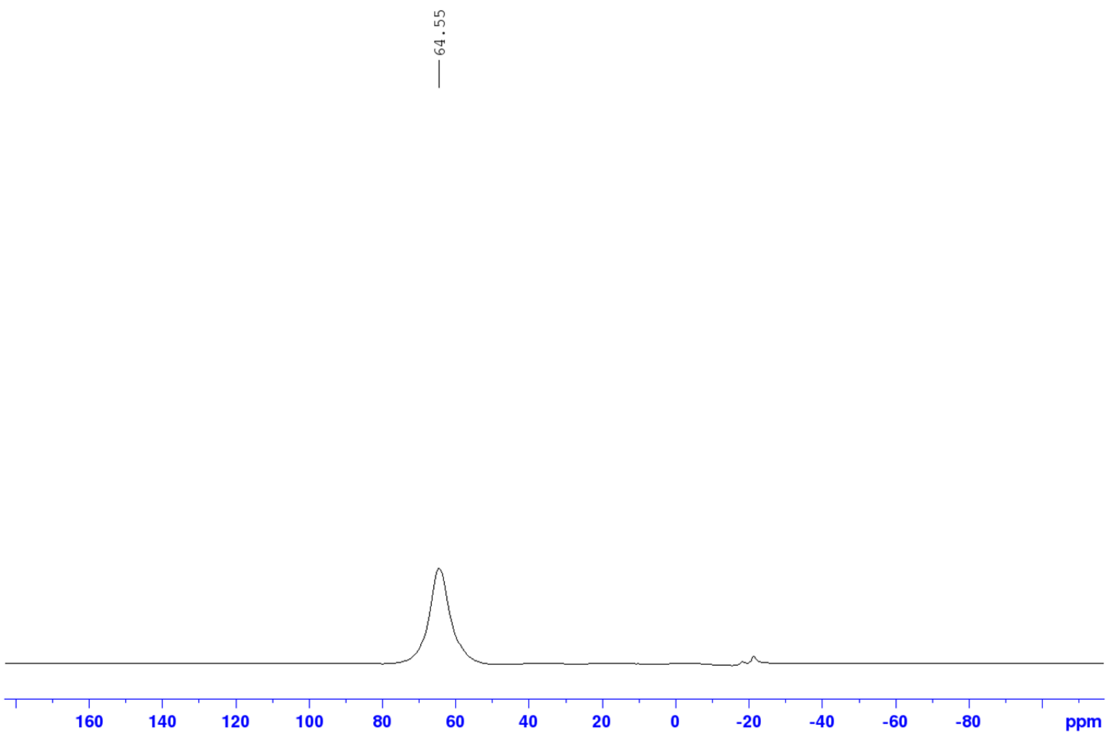


**Figure S18.** $^{11}B$ NMR spectrum of **7** in $C_6D_6$

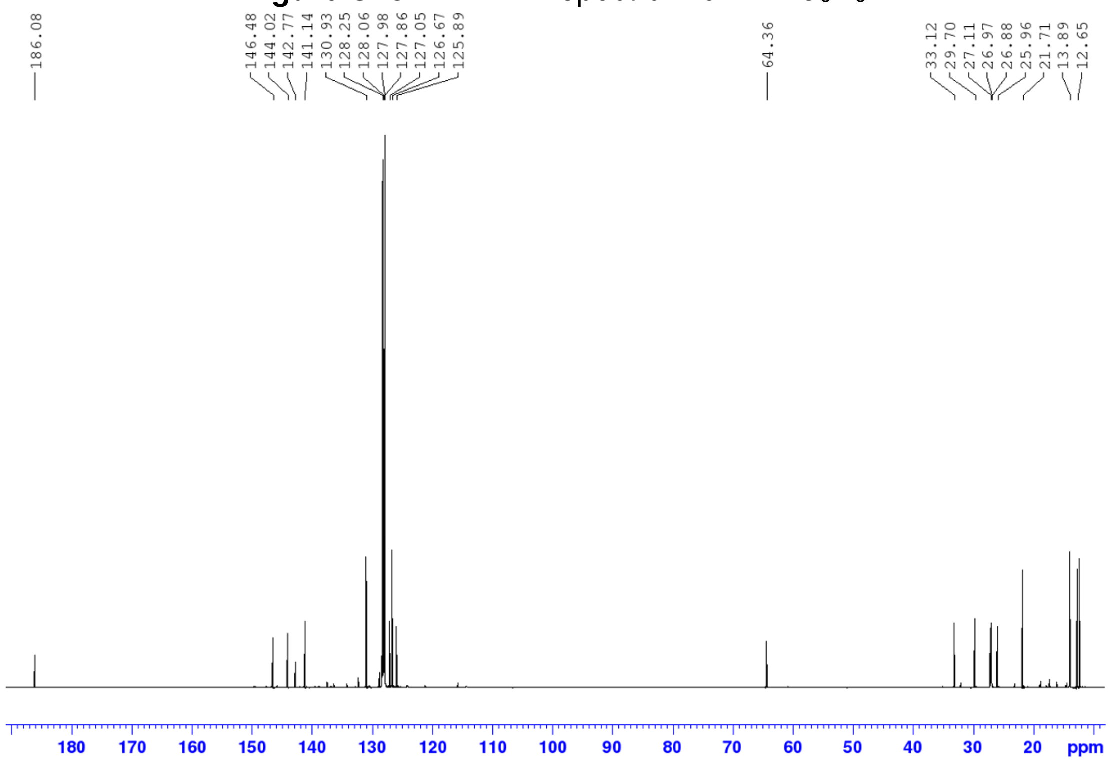


**Figure S19.** $^{1}H\{^{13}C\}$ NMR spectrum of **7** in $C_6D_6$

## Synthesis of 8

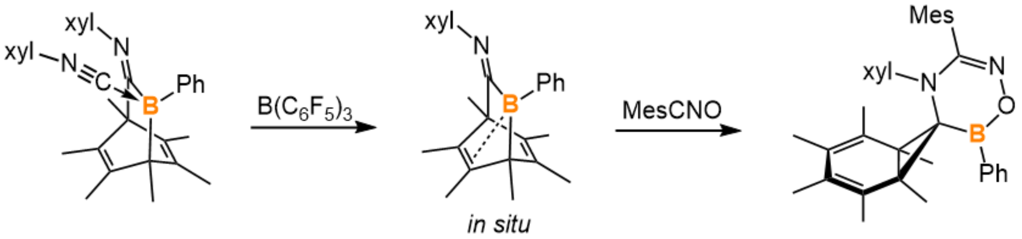


In a vial, compound **2** (217 mg, 0.42 mmol, 1.0 eq.) and $B(C_6F_5)_3$ (227.8 mg, 0.45 mmol, 1.05 eq.) were dissolved in 3 mL benzene. The solution was stirred for 5 min at 25 ºC. MesCNO (71.7 mg, 0.45 mmol, 1.05 eq.) was added to the stirring solution in one portion. The mixture was stirred for 5 min. All volatile materials were removed by vacuum. Hexanes (10 mL) was added and white solids crashed out. The solids were separated from the liquid via filtration and dried under a vacuum to afford the final product (175 mg, 0.32 mmol, 76.8%).

**$^1$H NMR** (500 MHz, $C_6D_6$): 0.61 (s, 3H), 0.98 (s, 3H), 1.11 (s, 3H),1.48 (s, 3H), 1.59 (s, 3H), 1.67 (s, 3H), 1.71 (s, 3H), 2.02 (s, 6H), 2.64 (s, 3H), 2.72 (s, 3H), 6.34 (m, 1H), 6.53 (m, 1H), 6.72 (s, 1H), 6.77 to 6.83 (m, 2H), 7.12 to 7.21 (m, 3H), 7.74 (d, *J* = 7.2 Hz, 2H). **$^{11}$B NMR** (161 MHz, $C_6D_6$): 42.1 (s). **$^{13}$C{$^1$H} NMR** (126 MHz, $C_6D_6$): 15.10 (s), 15.48 (s), 15.66 (s), 17.26 (s), 18.25 (s), 18.31 (s), 19.08 (s), 20.94 (s), 21.63 (s), 22.96 (s), 31.97 (s), 37.97 (s), 40.23 (s), 126.11 (s), 126.52 (s), 127.21 (s), 128.54 (s), 128.69 (s), 129.09 (s), 129.24 (s), 133.33 (s), 136.61 (s), 138.01 (s), 138.10 (s), 138.24 (s), 138.73 (s), 142.49 (s), 158.59 (s). **HRMS** (m/z): $[M+H]^+$ calcd. for $C_{37}H_{43}BN_2O$, 543.35467, found 543.35575.

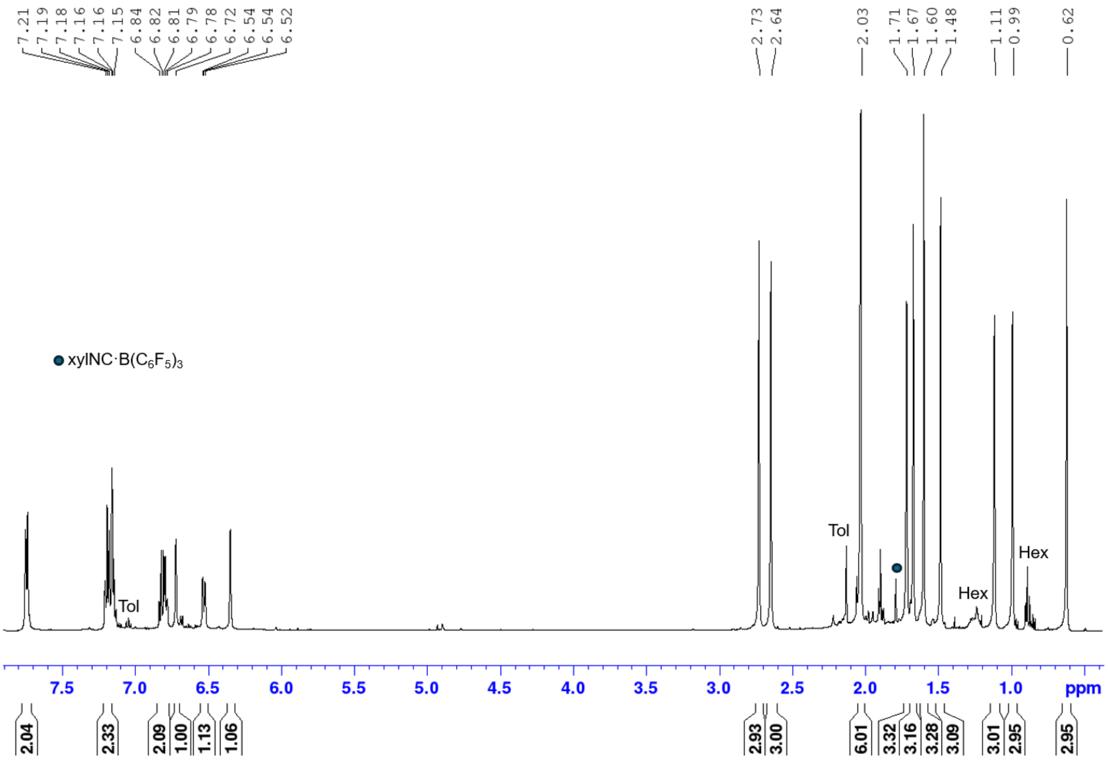


**Figure S20.** $^1$H NMR spectrum of **8** in $C_6D_6$

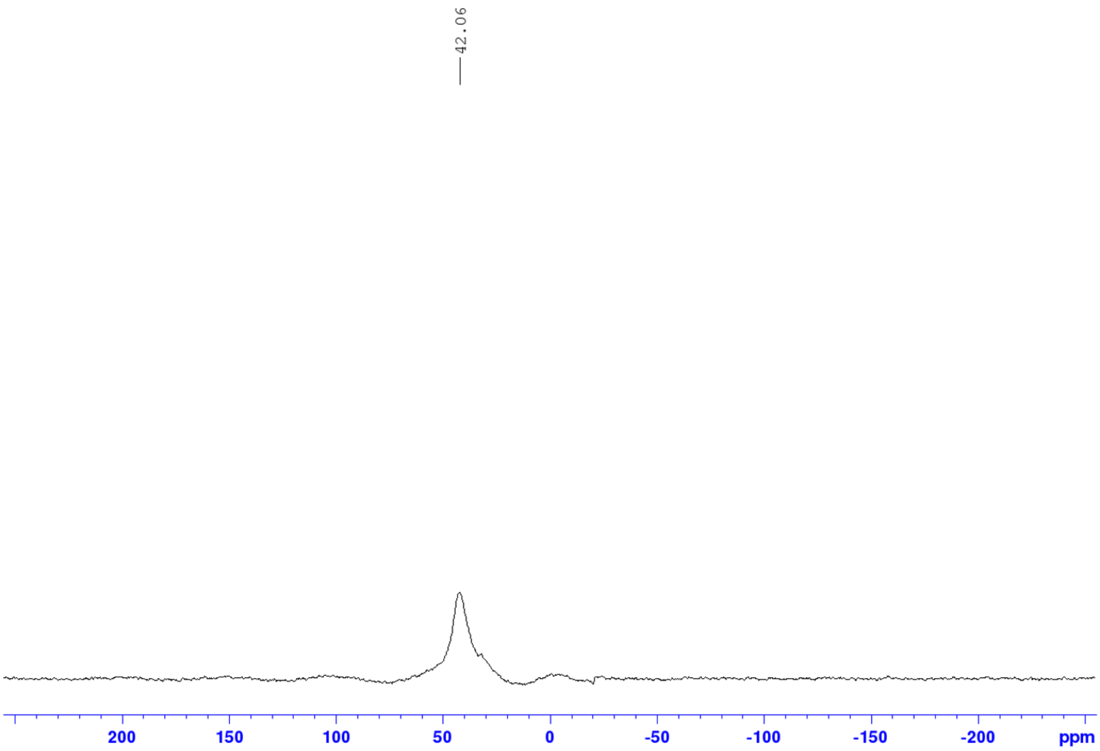


**Figure S21.** $^{11}$B NMR spectrum of **8** in $C_6D_6$

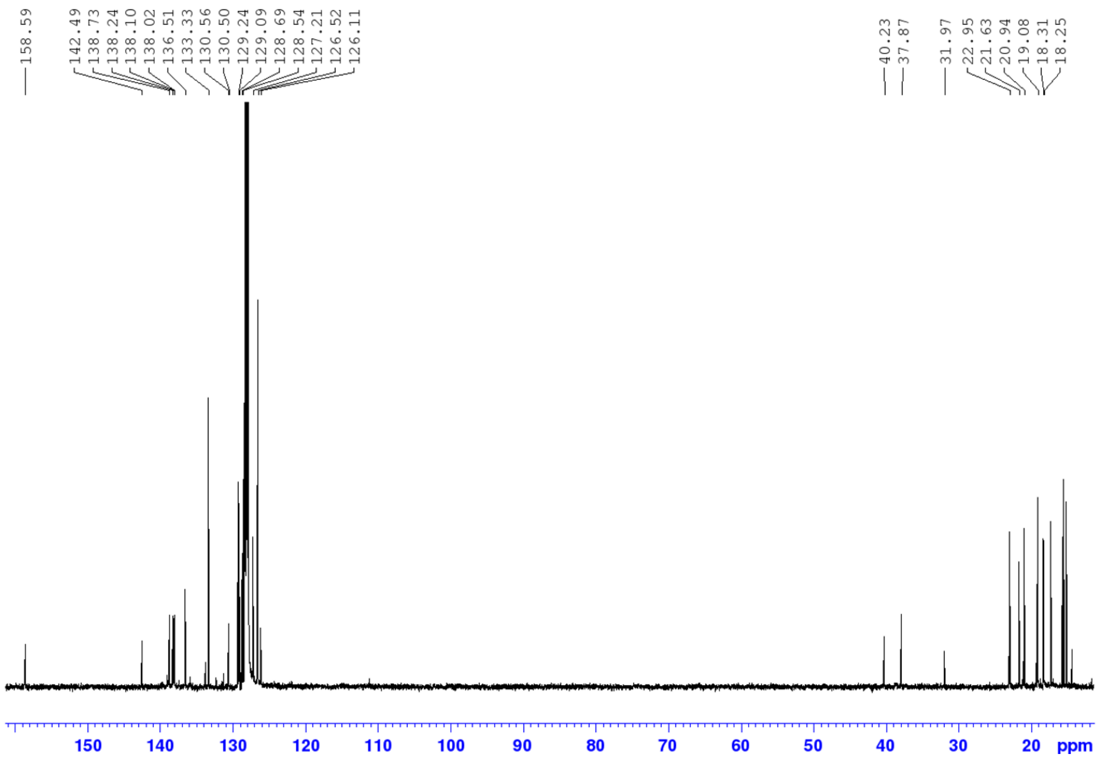


**Figure S22.** $^{13}C\{^{1}H\}$ NMR spectrum of **8** in $C_6D_6$

**Synthesis of 9:** In a vial, PhB($C_6Me_6$) (100 mg, 0.40 mmol, 1.0 eq.) and trimethylamine *N*-oxide (30 mg, 0.40 mmol, 1.0 eq.) were dissolved in THF (5 mL). The solution was stirred at room temperature for an hour, and all volatiles were removed under vacuum. The remaining solid was heated at 150 °C under vacuum for an hour to remove hexamethylbenzene, after which the solid was identified as triphenylboroxin (40 mg, 0.13 mmol, 97%). The NMR spectra of **9** are in accordance with those in the literature.[4]

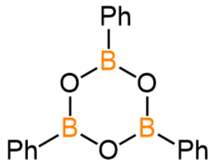


**$^1$H NMR** (500 MHz, $CDCl_3$): 7.52 (d, *J* = 7.2 Hz, 6H), 7.61 (t, *J* = 7.4 Hz, 3H), 8.25 (t, *J* = 7.4 Hz, 6H). **$^{11}$B NMR** (161 MHz, $CDCl_3$): 29.01 (s). **HRMS** (m/z): $[M+H]^+$ calcd. for $C_{18}H_{16}B_3O_3$, 313.13786; found 313.14245.

**Synthesis of 10:** In a Schlenk flask, PhB($C_6Me_6$) (100 mg, 0.40 mmol, 1.0 eq.) was dissolved in toluene (10 mL) and then $PhN_3$ (0.1 M in methyl *tert*-butyl ether) was added (2.0 mL, 2.0 mmol, 5.0 eq.). The solution was stirred and heated at 50 °C for 5 days. All volatile materials in the solution were removed under vacuum. The remaining solid was stirred with hexane (10 mL), and the liquid was removed by pipette to remove hexamethylbenzene. The solid was dissolved in toluene (approx. 2 mL) and added with hexane (approx. 1 mL) for recrystallization. The solution was stored at –35 °C to afford a white crystalline solid. The mother liquor was removed by filtration, and the solid was dried under vacuum (87 mg, 0.29 mmol, 73%).

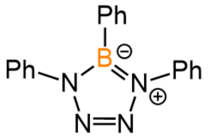


**$^1$H NMR** (500 MHz, $C_6D_6$): 6.91 (td, *J* = 7.5 Hz, 1.4, 2H), 6.99 (m, 4H), 7.02 (m, 2H), 7.08 (m, 1H), 7.17 to 7.19 (m, 2H), 7.45 (m, 4H). **$^{11}$B NMR** (161 MHz, $C_6D_6$): 25.36 (s). **$^{13}$C{$^1$H} NMR** (126 MHz, $C_6D_6$): 122.80 (s), 126.67 (s), 128.57 (s), 129.31 (s), 129.82 (s), 134.09 (s), 140.37 (s). **HRMS** (m/z): $[M+H]^+$ calcd. for $C_{18}H_{16}BN_4$, 299.14680; found 299.15183.

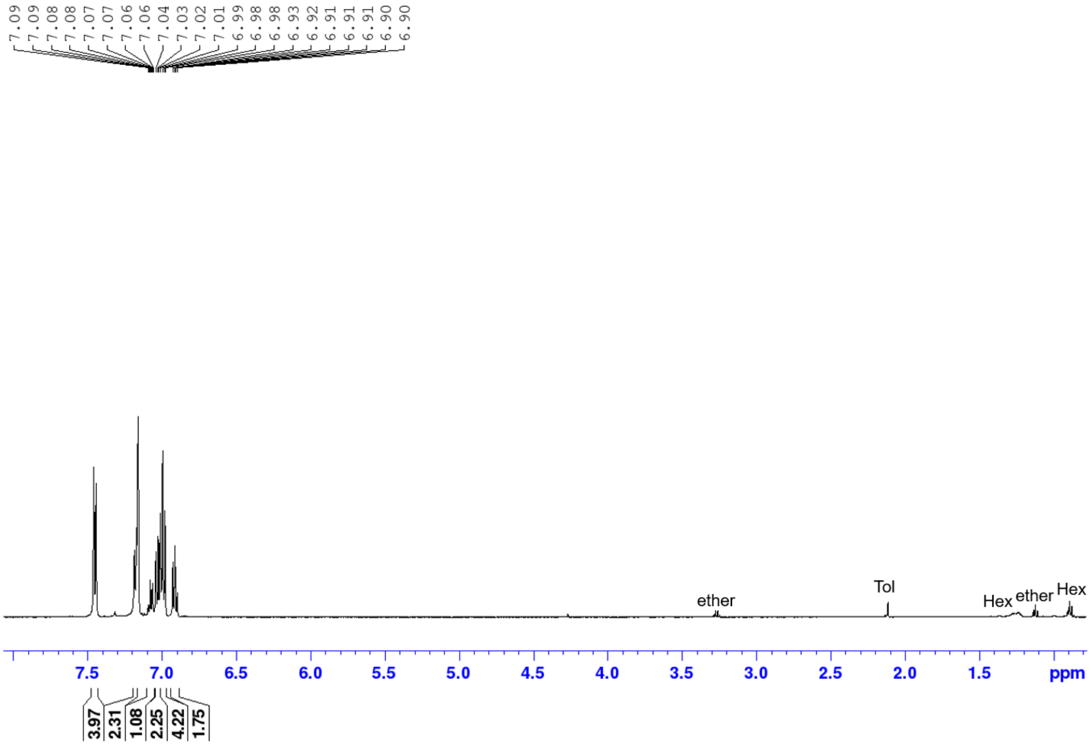


**Figure S23.** $^{1}$H NMR spectrum of **10** in $C_6D_6$

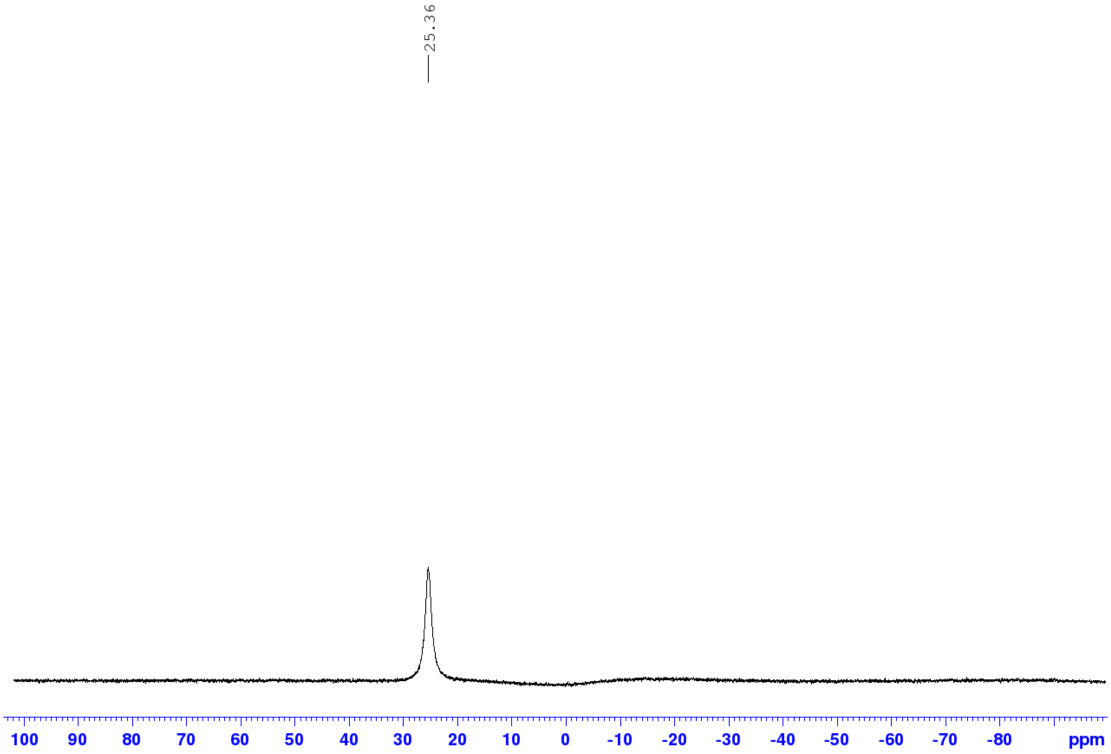


**Figure S24.** $^{11}$B NMR spectrum of **10** in $C_6D_6$

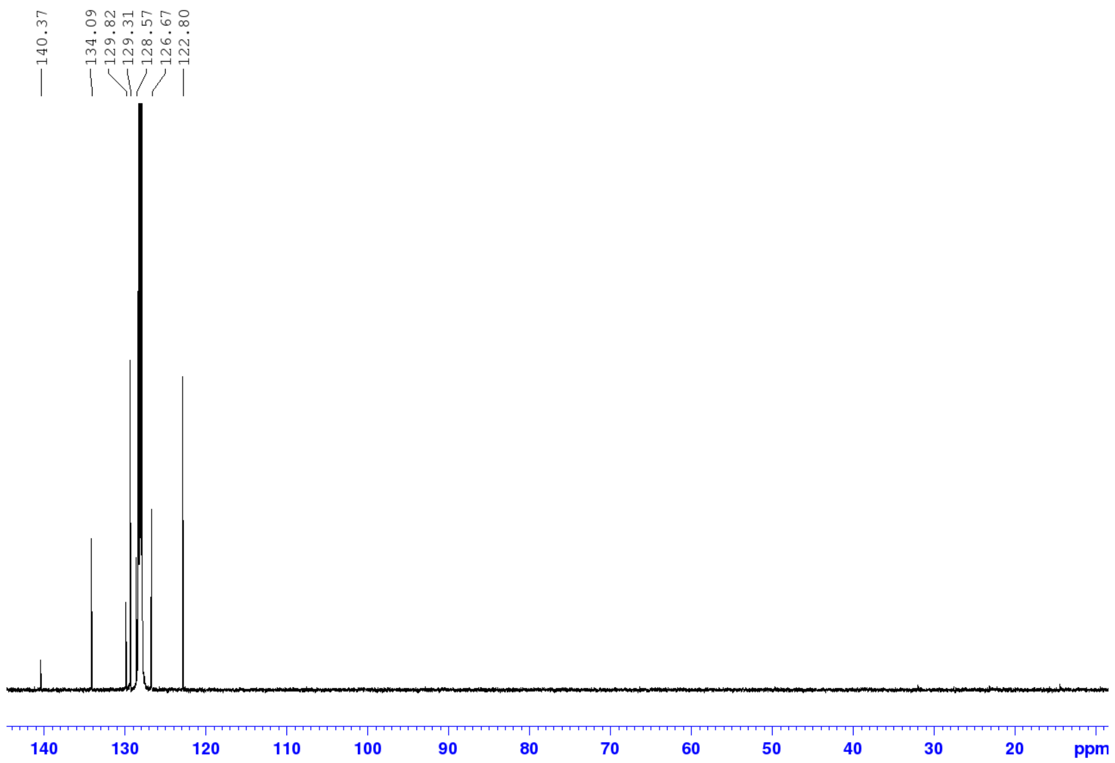


**Figure S25.** $^{13}C\{^1H\}$ NMR spectrum of **10** in $C_6D_6$

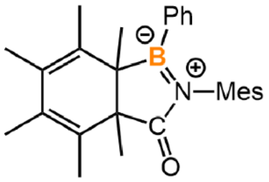


**Synthesis of 11:** $PhB(C_6Me_6)$ (100 mg, 0.40 mmol, 1.0 eq.) and mesityl isocyanate (MesNCO) (67.7 mg, 0.42 mmol, 1.05 eq.) were added to toluene (5 mL). The solution was stirred at 50 °C for 2 days. All volatile materials in the solution were removed under vacuum, and the remaining solid was washed with cold hexane (0.5×2 mL). The solid was crystallized from hexane/toluene to afford the final product as a white solid (102 mg, 0.25 mmol, 62%).

**$^1$H NMR** (500 MHz, $C_6D_6$): 1.21 (s, 3H), 1.31 (s, 3H), 1.66 (s, 3H), 1.72 (s, 3H), 1.81 (s, 6H), 1.90 (s, 3H), 2.06 (s, 3H), 2.31 (s, 3H), 6.57 (s, 1H), 6.80 (s, 3H), 6.99 (m, 3H), 7.34 (m, 2H). **$^{11}$B NMR** (161 MHz, $C_6D_6$): 53.46 (br. s). **$^{13}$C{$^1$H} NMR** (126 MHz, $C_6D_6$): 13.47 (s), 15.32 (s), 15.81 (s), 16.11 (s), 17.02 (s), 17.75 (s), 19.10 (s), 19.30 (s), 20.94 (s), 57.71 (s), 125.70 (s), 125,95 (s), 129.57 (s), 128.85 (s), 129.06 (s), 129.33 (s), 129.53 (s), 129.66 (s), 130.21 (s), 130.34 (s), 132.56 (s), 134.02 (s), 134.63 (s), 135.74 (s), 136.56 (s), 187.73 (s). **HRMS** (m/z): $[M+H]^+$ calcd. for $C_{28}H_{34}BN$, 395.27842; found 395.27338. **Elemental analysis**: calcd. for $C_{28}H_{34}BNO$, C, 81.75; H, 8.33; N, 3.40; found, C, 81.47; H, 8.28; N, 3.52.

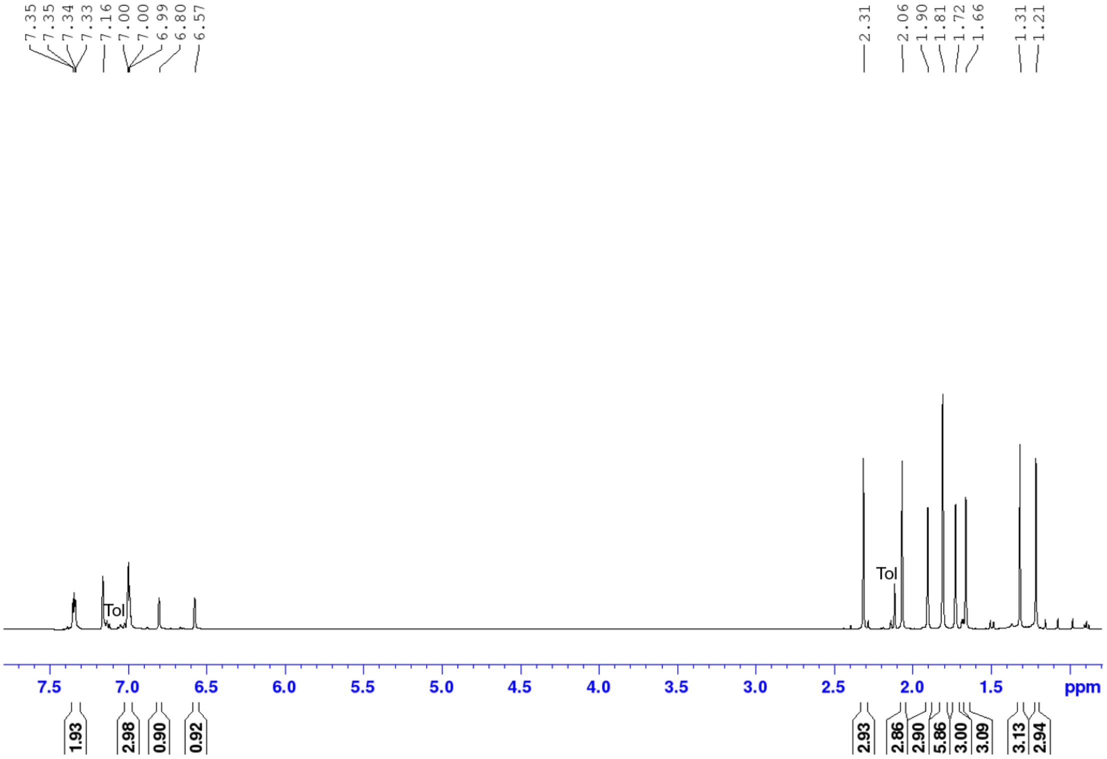


**Figure S26.** $^1$H NMR spectrum of **11** in $C_6D_6$

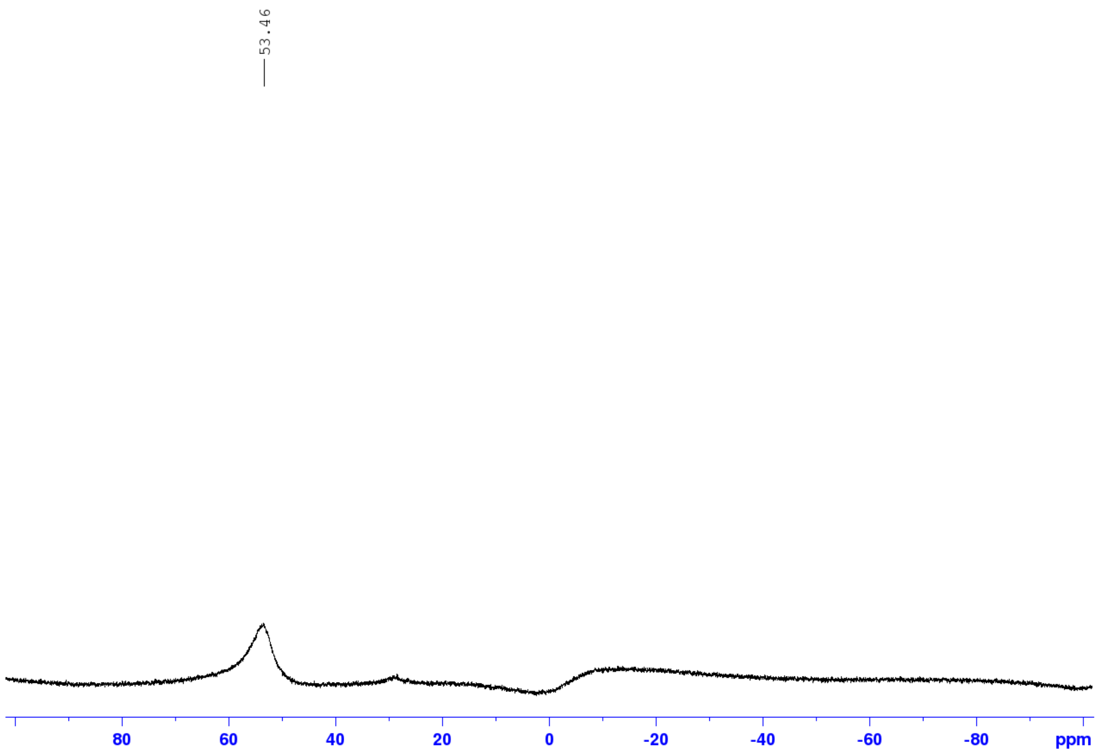


**Figure S27.** $^{11}B$ NMR spectrum of **11** in $C_6D_6$

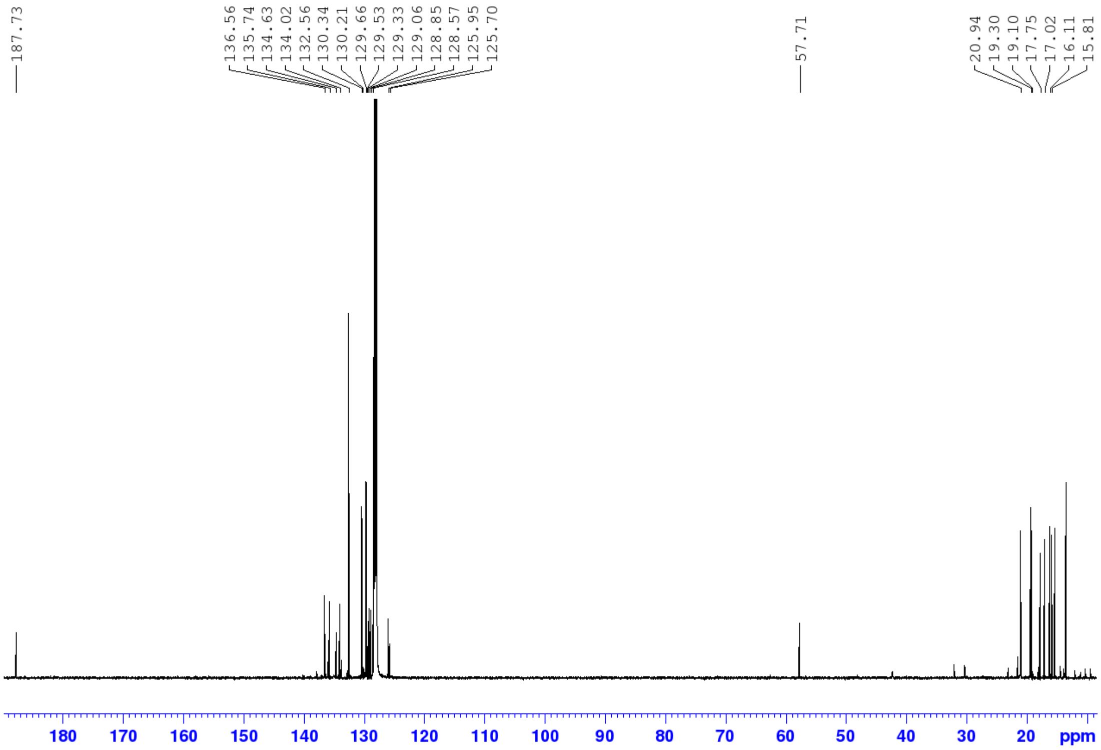


**Figure S28.** $^{13}C\{^1H\}$ NMR spectrum of **11** in $C_6D_6$

**Concentration dependence study of the reaction of 1 with 2,6-xylyl isocyanide**

In a vial, PhB($C_6Me_6$) (**1**) and 2,6-xylyl isocyanide (xylNC) were dissolved in toluene. The solution was stirred at room temperature for three days, and the color gradually turned yellow. All volatiles in the solution were removed under vacuum, and the remaining solids were redissolved in $C_6D_6$. The NMR yield of **3** was determined in each experiment, and using an increased equivalent of xylNC led to a notable enhancement in forming **3**.

**Table S1.** NMR yields of **3** in different experiments.

| No. of experiment | **1** | xylNC | toluene | **1**(mol): xylNC(mol) | NMR yields of **3** |
|---|---|---|---|---|---|
| 1 | 12.5 mg | 14 mg | 4 mL | 1:2 | 9% |
| 2 | 12.5 mg | 28 mg | 4 mL | 1:4 | 15% |
| 3 | 12.5 mg | 42 mg | 4 mL | 1:6 | 18% |
| 4 | 12.5 mg | 70 mg | 4 mL | 1:10 | 23% |

## Monitoring the fragmentation of 2

In a J Young NMR tube, compound **2** (25 mg, 0.05 mmol, 1.0 equiv) and acenaphthene (internal reference, 15.4 mg, 0.10 mmol, 1.0 equiv) were dissolved in d8-toluene (0.5 mL). The NMR tube was heated at 90 °C and the reaction was monitored by $^1$H NMR spectroscopy. The fragmentation of **2** is neither a first-order nor second-order kinetics.

**Table 2.** The concentration of $C_6Me_6$ (mol/L) at different times according to the integration of $C_6Me_6$ and acenaphthene.

| Time/min | Concentration of $C_6Me_6$ (mol/L) | Time/min | Concentration of $C_6Me_6$ (mol/L) |
|---|---|---|---|
| 0 | 0.0196 | 55 | 0.0696 |
| 8 | 0.0305 | 61 | 0.0726 |
| 15 | 0.0389 | 78 | 0.0765 |
| 27 | 0.0470 | 95 | 0.0801 |
| 32 | 0.0555 | 113 | 0.0831 |
| 38 | 0.0611 | 130 | 0.0845 |
| 43 | 0.0642 | 158 | 0.0860 |
| 49 | 0.0666 | 1000+ | 0.0897 |

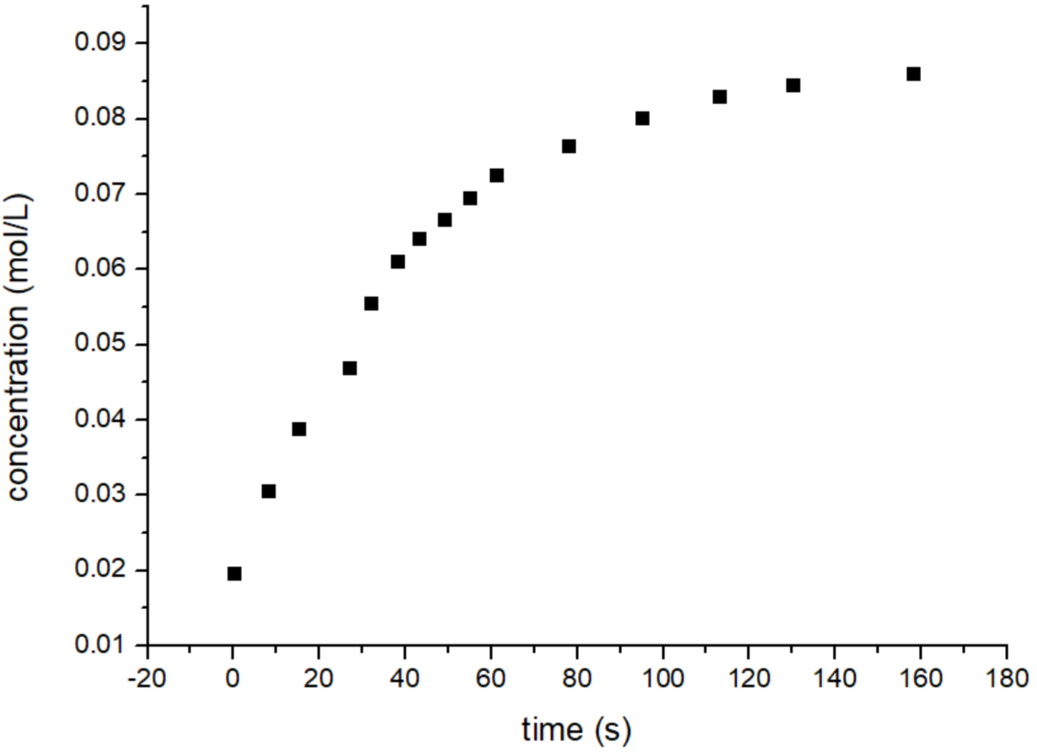


**Figure S29.** Plot of the concentration of $C_6Me_6$ (mol/L) at different times

In a J Young NMR tube, compound **2** (15 mg) was dissolved in d8-toluene (0.5 mL). The NMR tube was heated at 90 °C and the reaction was monitored

by $^{11}B$ NMR spectroscopy. It revealed an intermediate at δ = −17.0 ppm, comparable to the boraketenimine $TpB(CNMe)_2$.

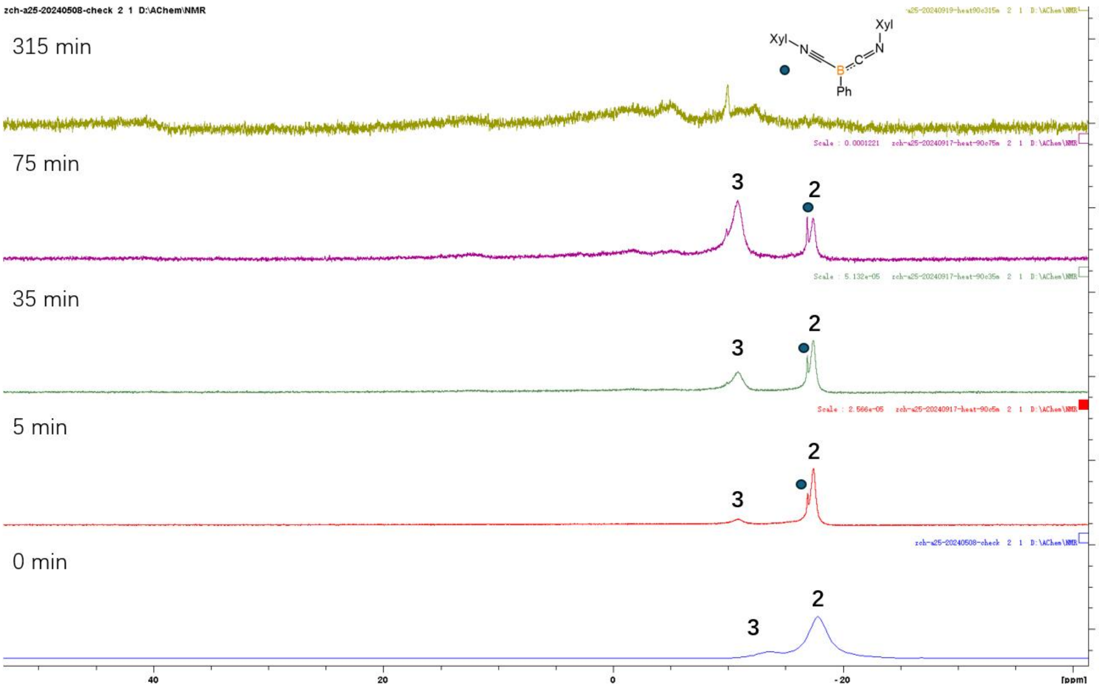


**Figure S30.** $^{11}B$ NMR of heating compound **2** in d8-toluene at different times

## Other attempted experiments

Treatment of **1** with CO

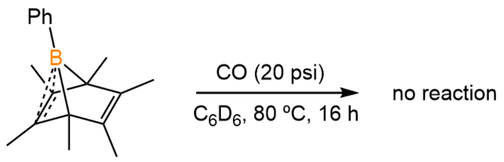


In a J. Young NMR tube, PhB($C_6Me_6$) (12.5 mg, 0.05 mmol, 1.0 equiv) was dissolved in $C_6D_6$ (0.5 mL). The solution was subjected to three freeze–pump–thaw cycles and then pressurized with CO (20 psi). It was heated at 80 °C for 16 hours. $^{11}$B NMR spectroscopy showed no change in the starting material, PhB($C_6Me_6$).

Attempted trapping of the oxoborane (PhB≡O)

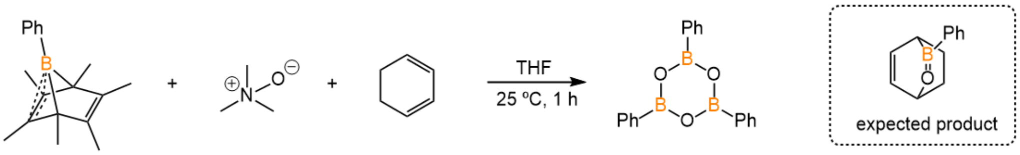


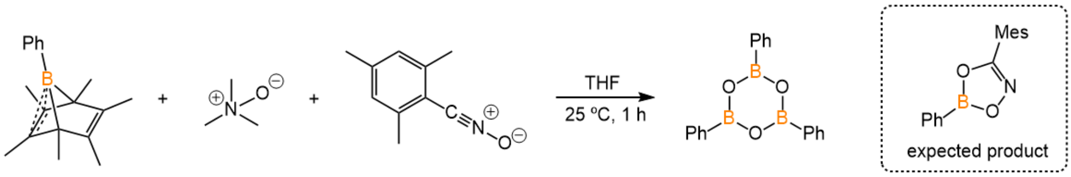


**Figure S31.** Attempted trapping of the oxoborane PhB≡O by cyclohexadiene and mesityl nitrile oxide, aiming to afford the corresponding cycloaddition product.

In a vial, PhB($C_6Me_6$) (12.5 mg, 0.05 mmol, 1.0 eq.), trimethylamine *N*-oxide (3.7 mg, 0.05 mmol, 1.0 eq.), and a trapping reagent (for $C_6H_8$, 80.0 mg, 0.50 mmol, 10.0 equiv; for MesCNO, 8.1 mg, 0.05 mmol, 1.0 equiv) were dissolved in THF (2 mL). The solution was stirred at room temperature for 1 hour, after which all volatiles were removed under vacuum. The remaining solid was redissolved in $C_6D_6$, and the major product was identified as triphenylboroxin by $^1$H NMR spectroscopy.

## Crystal structures

Low-temperature (100 K) diffraction data were collected on a Bruker-AXS X8 Kappa Duo diffractometer with IμS micro-sources, coupled to a Photon 3 CPAD detector for all structures. Mo $K_\alpha$ radiation ($\lambda$ = 0.71073 Å) was used for all the structures. Data reduction, scaling and absorption corrections were performed using SAINT (Bruker, V8.38A, 2013). The structure was solved with the XT structure solution program using the Intrinsic Phasing solution method[5] and by using Olex2[6] as the graphical interface. The model was refined with the ShelXL program[7] using Least Squares minimization. All non-hydrogen atoms were refined anisotropically. Hydrogen atoms were included in structure factor calculations. All hydrogen atoms were assigned to idealized geometric positions.

Crystallographic data have been deposited with the Cambridge Crystallographic Data as supplementary publication nos. CCDC-2299088 (**2**), 2299089 (**3**), 2299090 (**4**), 2299091 (**5**&**6**), 2299092 (**10**), 2299093 (**11**), 2416228 (**7**), 2416229 (**8**). These data can be obtained free of charge from The Cambridge Crystallographic Data Centre via Data https://www.ccdc.cam.ac.uk.

**Table 3.** Crystal data and structure refinement for **2**.

| | |
|---|---|
| Identification code | 2299088 |
| Empirical formula | $C_{36}H_{41}BN_2$ |
| Formula weight | 512.52 |
| Temperature/K | 100.00 |
| Crystal system | monoclinic |
| Space group | $P2_1/n$ |
| a/Å | 11.5762(6) |
| b/Å | 19.2982(11) |
| c/Å | 13.3269(7) |
| α/° | 90 |
| β/° | 99.970(2) |
| γ/° | 90 |
| Volume/Å$^3$ | 2932.3(3) |
| Z | 4 |
| $\rho_{calc}$g/cm$^3$ | 1.161 |
| μ/mm$^{-1}$ | 0.066 |
| F(000) | 1104.0 |
| Crystal size/mm$^3$ | 0.121 × 0.111 × 0.098 |
| Radiation | MoKα (λ = 0.71073) |
| 2Θ range for data collection/° | 3.752 to 55.132 |
| Index ranges | -15 ≤ h ≤ 14, -25 ≤ k ≤ 25, -17 ≤ l ≤ 17 |
| Reflections collected | 88037 |
| Independent reflections | 6755 [$R_{int}$ = 0.1793, $R_{sigma}$ = 0.0715] |

| | |
|---|---|
| Data/restraints/parameters | 6755/0/362 |
| Goodness-of-fit on $F^2$ | 1.002 |
| Final R indexes [I>=2σ (I)] | $R_1$ = 0.0440, $wR_2$ = 0.1089 |
| Final R indexes [all data] | $R_1$ = 0.0731, $wR_2$ = 0.1159 |
| Largest diff. peak/hole / e $Å^{-3}$ | 0.21/-0.25 |

**Table 4. Crystal data and structure refinement for 3.**

| | |
|---|---|
| Identification code | 2299089 |
| Empirical formula | $C_{45}H_{50}BN_3$ |
| Formula weight | 643.69 |
| Temperature/K | 100.00 |
| Crystal system | monoclinic |
| Space group | $P2_1/c$ |
| a/Å | 17.4580(13) |
| b/Å | 14.7741(11) |
| c/Å | 16.2688(12) |
| α/° | 90 |
| β/° | 104.417(2) |
| γ/° | 90 |
| Volume/$Å^3$ | 4064.0(5) |
| Z | 4 |
| $\rho_{calc}$g/cm$^3$ | 1.052 |
| μ/mm$^{-1}$ | 0.060 |
| F(000) | 1384.0 |
| Crystal size/mm$^3$ | 0.27 × 0.021 × 0.012 |
| Radiation | MoKα (λ = 0.71073) |
| 2Θ range for data collection/° | 3.66 to 50.05 |
| Index ranges | -20 ≤ h ≤ 20, -17 ≤ k ≤ 17, -19 ≤ l ≤ 18 |
| Reflections collected | 144514 |
| Independent reflections | 7184 [$R_{int}$ = 0.1206, $R_{sigma}$ = 0.0459] |
| Data/restraints/parameters | 7184/0/454 |
| Goodness-of-fit on $F^2$ | 1.021 |
| Final R indexes [I>=2σ (I)] | $R_1$ = 0.0593, $wR_2$ = 0.1312 |
| Final R indexes [all data] | $R_1$ = 0.0943, $wR_2$ = 0.1502 |
| Largest diff. peak/hole / e $Å^{-3}$ | 0.20/-0.23 |

**Table 5 Crystal data and structure refinement for 4.**

| | |
|---|---|
| Identification code | 2299090 |
| Empirical formula | $C_{78}H_{76}B_2N_6$ |
| Formula weight | 1119.06 |
| Temperature/K | 100.00 |
| Crystal system | triclinic |
| Space group | P-1 |
| a/Å | 10.8373(5) |
| b/Å | 12.1312(7) |

| | |
|---|---|
| c/Å | 13.6657(7) |
| α/° | 84.390(2) |
| β/° | 72.008(2) |
| γ/° | 68.497(2) |
| Volume/$Å^3$ | 1589.59(15) |
| Z | 1 |
| $ρ_{calc}$g/$cm^3$ | 1.169 |
| μ/$mm^{-1}$ | 0.068 |
| F(000) | 596.0 |
| Crystal size/$mm^3$ | 0.291 × 0.193 × 0.184 |
| Radiation | MoKα (λ = 0.71073) |
| 2Θ range for data collection/° | 3.134 to 50.05 |
| Index ranges | -12 ≤ h ≤ 12, -14 ≤ k ≤ 14, -16 ≤ l ≤ 16 |
| Reflections collected | 80309 |
| Independent reflections | 5618 [$R_{int}$ = 0.0357, $R_{sigma}$ = 0.0190] |
| Data/restraints/parameters | 5618/84/413 |
| Goodness-of-fit on $F^2$ | 1.021 |
| Final R indexes [I>=2σ (I)] | $R_1$ = 0.0604, $wR_2$ = 0.1492 |
| Final R indexes [all data] | $R_1$ = 0.0621, $wR_2$ = 0.1507 |
| Largest diff. peak/hole / e $Å^{-3}$ | 0.55/-0.40 |

**Table 6 Crystal data and structure refinement for 5&6.**

| | |
|---|---|
| Identification code | 2299091 |
| Empirical formula | $C_{72}H_{64}B_3F_{15}N_2$ |
| Formula weight | 1274.68 |
| Temperature/K | 100.00 |
| Crystal system | triclinic |
| Space group | P-1 |
| a/Å | 13.063(3) |
| b/Å | 15.942(3) |
| c/Å | 21.120(5) |
| α/° | 92.077(8) |
| β/° | 107.094(8) |
| γ/° | 109.231(7) |
| Volume/$Å^3$ | 3925.8(16) |
| Z | 2 |
| $ρ_{calc}$g/$cm^3$ | 1.078 |
| μ/$mm^{-1}$ | 0.087 |
| F(000) | 1320.0 |
| Crystal size/$mm^3$ | 0.24 × 0.12 × 0.032 |
| Radiation | MoKα (λ = 0.71073) |
| 2Θ range for data collection/° | 4.08 to 64.178 |
| Index ranges | -19 ≤ h ≤ 19, -23 ≤ k ≤ 23, -31 ≤ l ≤ 31 |
| Reflections collected | 337344 |
| Independent reflections | 27270 [$R_{int}$ = 0.0762, $R_{sigma}$ = 0.0372] |

| | |
|---|---|
| Data/restraints/parameters | 27270/328/1015 |
| Goodness-of-fit on $F^2$ | 1.020 |
| Final R indexes [I>=2σ (I)] | $R_1$ = 0.0713, $wR_2$ = 0.1747 |
| Final R indexes [all data] | $R_1$ = 0.0920, $wR_2$ = 0.1897 |
| Largest diff. peak/hole / e Å$^{-3}$ | 0.41/-0.35 |

**Table 7 Crystal data and structure refinement for 10.**

| | |
|---|---|
| Identification code | 2299092 |
| Empirical formula | $C_{35}H_{44}BN$ |
| Formula weight | 489.52 |
| Temperature/K | 100.00 |
| Crystal system | monoclinic |
| Space group | $P2_1/n$ |
| a/Å | 10.2681(2) |
| b/Å | 17.8570(4) |
| c/Å | 15.4419(3) |
| α/° | 90 |
| β/° | 100.3020(10) |
| γ/° | 90 |
| Volume/Å$^3$ | 2785.74(10) |
| Z | 4 |
| $\rho_{calc}$g/cm$^3$ | 1.167 |
| μ/mm$^{-1}$ | 0.066 |
| F(000) | 1064.0 |
| Crystal size/mm$^3$ | 0.098 × 0.078 × 0.065 |
| Radiation | MoKα (λ = 0.71073) |
| 2Θ range for data collection/° | 4.424 to 60.128 |
| Index ranges | -14 ≤ h ≤ 14, -25 ≤ k ≤ 25, -21 ≤ l ≤ 19 |
| Reflections collected | 32087 |
| Independent reflections | 7898 [$R_{int}$ = 0.0325, $R_{sigma}$ = 0.0277] |
| Data/restraints/parameters | 7898/0/342 |
| Goodness-of-fit on $F^2$ | 1.072 |
| Final R indexes [I>=2σ (I)] | $R_1$ = 0.0457, $wR_2$ = 0.1124 |
| Final R indexes [all data] | $R_1$ = 0.0521, $wR_2$ = 0.1172 |
| Largest diff. peak/hole / e Å$^{-3}$ | 0.39/-0.23 |

**Table 8 Crystal data and structure refinement for 11.**

| | |
|---|---|
| Identification code | 2299093 |
| Empirical formula | $C_{37}H_{43}BN_2O$ |
| Formula weight | 542.54 |
| Temperature/K | 100.00 |
| Crystal system | monoclinic |
| Space group | C2/c |
| a/Å | 20.640(2) |
| b/Å | 13.099(2) |

| | |
|---|---|
| c/Å | 22.222(3) |
| α/° | 90 |
| β/° | 96.787(7) |
| γ/° | 90 |
| Volume/Å$^3$ | 5966.1(16) |
| Z | 8 |
| $\rho_{calc}$g/cm$^3$ | 1.208 |
| μ/mm$^{-1}$ | 0.071 |
| F(000) | 2336.0 |
| Crystal size/mm$^3$ | 0.056 × 0.042 × 0.024 |
| Radiation | MoKα (λ = 0.71073) |
| 2Θ range for data collection/° | 3.69 to 59.156 |
| Index ranges | -28 ≤ h ≤ 28, -18 ≤ k ≤ 18, -30 ≤ l ≤ 30 |
| Reflections collected | 198194 |
| Independent reflections | 8370 [$R_{int}$ = 0.0814, $R_{sigma}$ = 0.0264] |
| Data/restraints/parameters | 8370/0/381 |
| Goodness-of-fit on F$^2$ | 1.038 |
| Final R indexes [I>=2σ (I)] | $R_1$ = 0.0529, w$R_2$ = 0.1245 |
| Final R indexes [all data] | $R_1$ = 0.0787, w$R_2$ = 0.1426 |
| Largest diff. peak/hole / e Å$^{-3}$ | 0.32/-0.24 |

**Table 9 Crystal data and structure refinement 7.**

| | |
|---|---|
| Identification code | 2416228 |
| Empirical formula | $C_{18}H_{15}BN_4$ |
| Formula weight | 298.15 |
| Temperature/K | 100.00 |
| Crystal system | monoclinic |
| Space group | P2$_1$/n |
| a/Å | 5.7579(4) |
| b/Å | 15.6788(11) |
| c/Å | 16.3799(11) |
| α/° | 90 |
| β/° | 94.763(3) |
| γ/° | 90 |
| Volume/Å$^3$ | 1473.62(18) |
| Z | 4 |
| $\rho_{calc}$g/cm$^3$ | 1.344 |
| μ/mm$^{-1}$ | 0.082 |
| F(000) | 624.0 |
| Crystal size/mm$^3$ | 0.087 × 0.016 × 0.008 |
| Radiation | MoKα (λ = 0.71073) |
| 2Θ range for data collection/° | 3.602 to 55.752 |
| Index ranges | -7 ≤ h ≤ 7, -20 ≤ k ≤ 20, -21 ≤ l ≤ 21 |
| Reflections collected | 34337 |
| Independent reflections | 3526 [$R_{int}$ = 0.0595, $R_{sigma}$ = 0.0318] |

| | |
|---|---|
| Data/restraints/parameters | 3526/0/208 |
| Goodness-of-fit on $F^2$ | 1.050 |
| Final R indexes [I>=2σ (I)] | $R_1$ = 0.0469, $wR_2$ = 0.0996 |
| Final R indexes [all data] | $R_1$ = 0.0722, $wR_2$ = 0.1141 |
| Largest diff. peak/hole / e $Å^{-3}$ | 0.21/-0.23 |

**Table 10 Crystal data and structure refinement for 8.**

| | |
|---|---|
| Identification code | 2416229 |
| Empirical formula | $C_{28}H_{34}BNO$ |
| Formula weight | 411.37 |
| Temperature/K | 100.00 |
| Crystal system | monoclinic |
| Space group | $P2_1/c$ |
| a/Å | 11.0019(8) |
| b/Å | 13.4954(11) |
| c/Å | 16.5173(12) |
| α/° | 90 |
| β/° | 100.269(3) |
| γ/° | 90 |
| Volume/$Å^3$ | 2413.1(3) |
| Z | 4 |
| $ρ_{calc}g/cm^3$ | 1.132 |
| μ/$mm^{-1}$ | 0.067 |
| F(000) | 888.0 |
| Crystal size/$mm^3$ | 0.249 × 0.246 × 0.211 |
| Radiation | MoKα (λ = 0.71073) |
| 2Θ range for data collection/° | 3.762 to 50.054 |
| Index ranges | -13 ≤ h ≤ 13, -16 ≤ k ≤ 16, -19 ≤ l ≤ 19 |
| Reflections collected | 75187 |
| Independent reflections | 4255 [$R_{int}$ = 0.0338, $R_{sigma}$ = 0.0121] |
| Data/restraints/parameters | 4255/0/289 |
| Goodness-of-fit on $F^2$ | 1.051 |
| Final R indexes [I>=2σ (I)] | $R_1$ = 0.0475, $wR_2$ = 0.1246 |
| Final R indexes [all data] | $R_1$ = 0.0497, $wR_2$ = 0.1268 |
| Largest diff. peak/hole / e $Å^{-3}$ | 0.31/-0.26 |

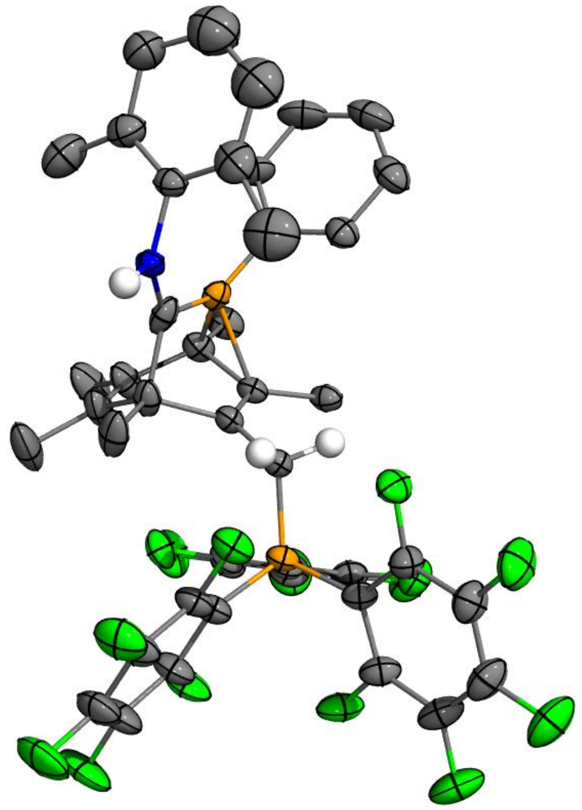

**Figure S32.** Single crystal structure of **6**. Partial hydrogen atoms have been omitted for clarity. Thermal ellipsoids are drawn at 50 % probability level. The 2,6-dimethylphenyl group has a two-fold disorder and only one part is displayed.

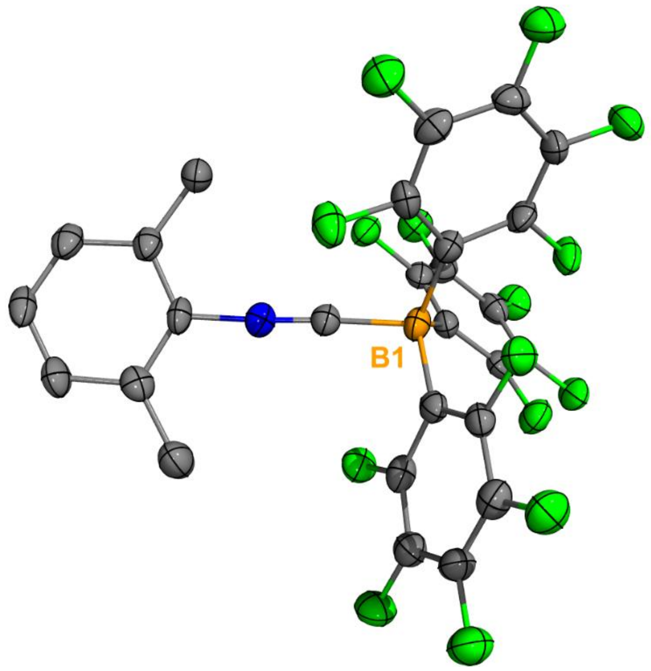

**Figure S33.** Single crystal structure of BCF· xylNC. Hydrogen atoms have been omitted for clarity. Thermal ellipsoids are drawn at 50 % probability level. The quality of the crystal structure was not publishable, but it displayed its atom connectivity.

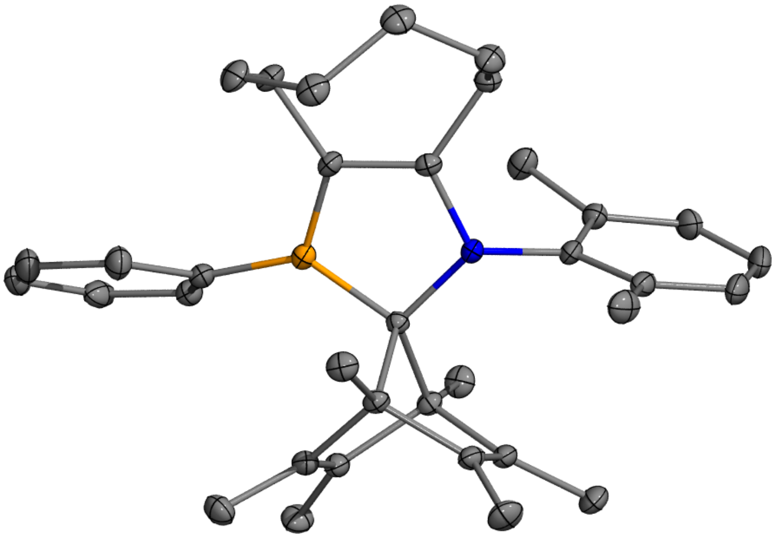

**Figure S34.** Single crystal structure of **7**. Hydrogen atoms have been omitted for clarity. Thermal ellipsoids are drawn at 50 % probability level.

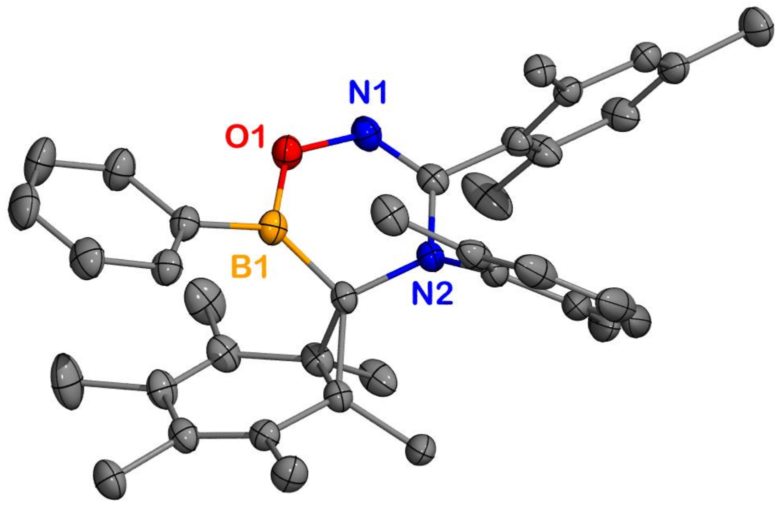


**Figure S35.** Single crystal structure of **8**. Hydrogen atoms have been omitted for clarity. Thermal ellipsoids are drawn at 50 % probability level.

## Computational Studies

Unless otherwise indicated, all calculations were performed with Gaussian 09 program[8]. The geometry optimizations and frequency calculation were performed at M06-2X/6-311G** level of theory,[9] incorperating D3 dispersion correction.[10] Frequency calculations were performed to confirm a transition state has only one imaginary frequency, while a local minimum has no imaginary frequency. Intrinsic reaction coordinate (IRC) calculations[11] were also carried out to further confirm that transition states can link the relevant local minima. All geometry optimizations, frequency calculations, and electronic energy calculations applied the SMD solvation model (in toluene).[12] Cartesian coordinates from all structures are compiled in xyz_all.xyz file which could be found at https://pubs.acs.org.

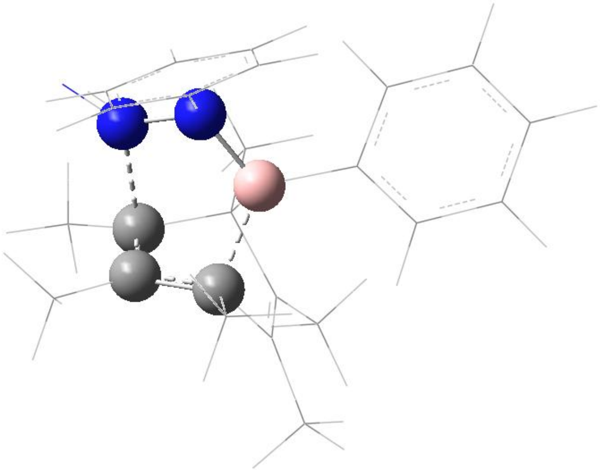

**Figure S36.** Optimized transition state $\mathbf{TS_1^N}$

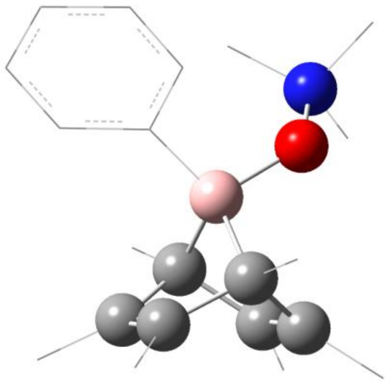

**Figure S37.** Optimized intermediate $\mathbf{I^O}$

**Table S11.** Computed energies for the formation of **4** in toluene. Computed Electronic Energy (EE, Hartree), total Gibbs free energies ($G_T$, Hartree), relative electronic energies ($\Delta E_2$, kcal/mol), relative Gibbs free energies ($\Delta G_T$, kcal/mol).

| | EE | $G_T$ | $\Delta E_2$ | $\Delta G_T$ |
|---|---|---|---|---|
| **2** | -1530.631257 | -1530.014492 | | |
| $C_6Me_6$ | -468.038768 | -467.807463 | | |
| **$TS_1^C$** | -1530.580166 | -1529.968578 | 32.06 | 28.81 |
| **$I^C$** | -1062.561761 | -1062.210755 | 19.28 | -2.34 |
| **$TS_2^C$** | -2125.109132 | -2124.376125 | 28.31 | 26.14 |
| **$II^C$** | -2125.177367 | -2124.436301 | -14.51 | -11.62 |
| **$TS_3^C$** | -2125.152378 | -2124.412248 | 1.17 | 3.47 |
| **$III^C$** | -2125.19659 | -2124.458484 | -26.57 | -25.54 |
| **$TS_4^C$** | -2125.162829 | -2124.422518 | -5.38 | -2.97 |
| **$IV^C$** | -2125.205409 | -2124.460955 | -32.10 | -27.09 |

**Table S12**. Computed energies for the formation of **9** in toluene solution. Computed Electronic Energy (EE, Hartree), total Gibbs free energies ($G_T$, Hartree), relative electronic energies ($\Delta E_2$, kcal/mol), relative Gibbs free energies ($\Delta G_T$, kcal/mol).

| | EE | $G_T$ | $\Delta E$ | $\Delta G_T$ |
|---|---|---|---|---|
| **1** | -724.484809 | -724.166413 | | |
| $Me_3NO$ | -249.579783 | -249.481419 | | |
| $C_6Me_6$ | -468.038768 | -467.807463 | | |
| $Me_3N$ | -174.430098 | -174.336127 | | |
| **$TS_1^O$** | -974.07019 | -973.633506 | -3.51 | 8.99 |
| **$I^O$** | -974.092381 | -973.651687 | -17.44 | -2.42 |
| **$TS_2^O$** | -974.056208 | -973.619884 | 5.26 | 17.54 |
| $Me_3N$+**$II^O$** | -974.182228 | -973.764981 | -73.82 | -73.51 |
| **$II^O$** | -799.75213 | -799.428854 | | |
| **$TS_3^O$** | -799.729124 | -799.407222 | -59.38 | -59.94 |
| PhBO+$C_6Me_6$ | -799.776054 | -799.476888 | -88.83 | -103.65 |
| PhBO | -331.737286 | -331.669425 | | |

**Table S13**. Computed energies for the formation of **10** in toluene solution. Computed Electronic Energy (EE, Hartree), total Gibbs free energies ($G_T$, Hartree), relative electronic energies ($\Delta E_2$, kcal/mol), relative Gibbs free energies ($\Delta G_T$, kcal/mol).

| | EE | $G_T$ | $\Delta E$ | $\Delta G_T$ |
|---|---|---|---|---|
| **1** | -724.484809 | -724.166413 | | |
| $PhN_3$ | -395.772671 | -395.700332 | | |
| $C_6Me_6$ | -468.038768 | -467.807463 | | |
| $N_2$ | -109.513587 | -109.526265 | | |
| **$I^N$** | -1120.253215 | -1119.83847 | 2.68 | 17.74 |
| **$TS_1^N$** | -1120.234121 | -1119.82096 | 14.66 | 28.73 |
| **$II^N$** | -1120.306872 | -1119.890283 | -30.99 | -14.77 |

| | | | | |
|---|---|---|---|---|
| **$TS_2^N$** | -1120.297588 | -1119.884628 | -25.17 | -11.22 |
| $N_2$+PhBNPh +$C_6Me_6$ | -1120.394568 | -1120.02437 | -86.02 | -98.91 |
| PhBNPh | -542.842213 | -542.690642 | | |
| **Nitrene Insertion Pathway** | | | | |
| **$TS_3^N$** | -1120.204716 | -1119.79432 | 33.11 | 45.45 |
| **$III^N$**+$N_2$ | -1120.392428 | -1119.995371 | -84.68 | -80.71 |
| **$III^N$** | -1010.878841 | -1010.469106 | | |

## Reference

[1] P. J. Fagan, E. G. Burns and J. C. Calabrese, *J. Am. Chem. Soc.* **1988**, *110*, 2979–2981.

[2] D. A. Roberts, B. S. Pilgrim, G. Sirvinskaite, T. K. Ronson and J. R. Nitschke, *J. Am. Chem. Soc.* **2018**, *140*, 9616-9623.

[3] G. Zhao, L. Liang, C. H. E. Wen and R. Tong, *Org. Lett.* **2019**, *21*, 315-319.

[4] Z. Alassad, A. Nandi, S. Kozuch and A. Milo, *J. Am. Chem. Soc.* **2023**, *145*, 89-98.

[5] G. M. Sheldrick, *Acta Cryst. A* **2015**, *71*, 3–8.

[6] O. V. Dolomanov, L. J. Bourhis, R. J. Gildea, J. A. K. Howard and H. Puschmann, *J. Appl. Cryst.* **2009**, *42*, 339–341.

[7] G. M. Sheldrick, *Acta Cryst. A* **2008**, *64*, 112–122.

[8] M. J. Frisch, G. W. Trucks, H. B. Schlegel, G. E. Scuseria, M. A. Robb, J. R. Cheeseman, G. Scalmani, V. Barone, G. A. Petersson, H. Nakatsuji, X. Li, M. Caricato, A. Marenich, J. Bloino, B. G. Janesko, R. Gomperts, B. Mennucci, H. P. Hratchian, J. V. Ortiz, A. F. Izmaylov, J. L. Sonnenberg, D. Williams-Young, F. Ding, F. Lipparini, F. Egidi, J. Goings, B. Peng, A. Petrone, T. Henderson, D. Ranasinghe, V. G. Zakrzewski, J. Gao, N. Rega, G. Zheng, W. Liang, M. Hada, M. Ehara, K. Toyota, R. Fukuda, J. Hasegawa, M. Ishida, T. Nakajima, Y. Honda, O. Kitao, H. Nakai, T. Vreven, K. Throssell, J. A. Montgomery, J. E. P. Jr., F. Ogliaro, M. Bearpark, J. J. Heyd, E. Brothers, K. N. Kudin, V. N. Staroverov, T. Keith, R. Kobayashi, J. Normand, K. Raghavachari, A. Rendell, J. C. Burant, S. S. Iyengar, J. Tomasi, M. Cossi, J. M. Millam, M. Klene, C. Adamo, R. Cammi, J. W. Ochterski, R. L. Martin, K. Morokuma, O. Farkas, J. B. Foresman and D. J. Fox, *Gaussian, Inc., Wallingford CT* **2016**.

[9] a) J.-D. Chai and M. Head-Gordon, *Phys. Chem. Chem. Phys.* **2008**, *10*, 6615; b) A. D. McLean and G. S. Chandler, *J. Chem. Phys.* **1980**, *72*, 5639–5648; c) R. Krishnan, J. S. Binkley, R. Seeger and J. A. Pople, *J. Chem. Phys.* **1980**, *72*, 650–654; d) M. M. Francl, W. J. Pietro, W. J. Hehre, J. S. Binkley, M. S. Gordon, D. J. Defrees and J. A. Pople, *J. Chem. Phys.* **1982**, *77*, 3654–3665; e) L. A. Curtiss, M. P. McGrath, J. P. Blaudeau, N. E. Davis, R. C. Binning and L. Radom, *J. Chem. Phys.* **1995**, *103*, 6104–6113; f) Y. Zhao and D. G. Truhlar, *Theor. Chem. Acc.* **2008**, *120*, 215–241.

[10] S. Grimme, J. Antony, S. Ehrlich and H. Krieg, *J. Chem. Phys.* **2010**, *132*, 154104.

[11] a) K. Fukui, *J. Phys. Chem.* **1970**, *74*, 4161–4163; b) K. Fukui, *Acc. Chem. Res.* **1981**, *14*, 363–368.

[12] A. V. Marenich, C. J. Cramer and D. G. Truhlar, *J. Phys. Chem. B* **2009**, *113*, 6378–6396.
